# [^18^F]FDG PET radiomics to predict disease-free survival in cervical cancer: a multi-scanner/center study with external validation

**DOI:** 10.1007/s00259-021-05303-5

**Published:** 2021-03-26

**Authors:** Marta Ferreira, Pierre Lovinfosse, Johanne Hermesse, Marjolein Decuypere, Caroline Rousseau, François Lucia, Ulrike Schick, Caroline Reinhold, Philippe Robin, Mathieu Hatt, Dimitris Visvikis, Claire Bernard, Ralph T. H. Leijenaar, Frédéric Kridelka, Philippe Lambin, Patrick E. Meyer, Roland Hustinx

**Affiliations:** 1 GIGA-CRC in vivo Imaging, University of Liège, GIGA, Avenue de l’Hôpital 11, 4000 Liege, Belgium; 2 Division of Nuclear Medicine and Oncological Imaging, University Hospital of Liège, Liège, Belgium; 3 Department of Radiation Oncology, Liège University Hospital, Liège, Belgium; 4 Division of Oncological Gynecology, University Hospital of Liège, Liège, Belgium; 5 Université de Nantes, CNRS, Inserm, CRCINA, F-44000 Nantes, France; 6 ICO René Gauducheau, F-44800 Saint-Herblain, France; 7 Radiation Oncology Department, University Hospital, Brest, France; 8 LaTIM, INSERM, UMR 1101, Univ Brest, Brest, France; 9 Department of Radiology, McGill University Health Centre (MUHC), Montreal, Canada; 10 Department of Nuclear Medicine and EA3878, Brest University Hospital, University of Brest, Brest, France; 11 Oncoradiomics SA, Clos Chanmurly 13, 4000 Liège, Belgium; 12 The-D Lab, Precision Medicine, GROW-School for Oncology and Developmental Biology, Maastricht University Medical Centre, Maastricht, Netherlands; 13 Department of Radiology and Nuclear Medicine, Maastricht University Medical Centre, Maastricht, The Netherlands; 14 Bioinformatics and Systems Biology Lab, University of Liège, Liège, Belgium

**Keywords:** Radiomics, [^18^F]FDG PET/CT, Cervical cancer, Disease-free survival, Machine learning

## Abstract

**Purpose:**

To test the performances of native and tumour to liver ratio (TLR) radiomic features extracted from pre-treatment 2-[^18^F] fluoro-2-deoxy-D-glucose ([^18^F]FDG) PET/CT and combined with machine learning (ML) for predicting cancer recurrence in patients with locally advanced cervical cancer (LACC).

**Methods:**

One hundred fifty-eight patients with LACC from multiple centers were retrospectively included in the study. Tumours were segmented using the Fuzzy Local Adaptive Bayesian (FLAB) algorithm. Radiomic features were extracted from the tumours and from regions drawn over the normal liver. Cox proportional hazard model was used to test statistical significance of clinical and radiomic features. Fivefold cross validation was used to tune the number of features. Seven different feature selection methods and four classifiers were tested. The models with the selected features were trained using bootstrapping and tested in data from each scanner independently. Reproducibility of radiomics features, clinical data added value and effect of ComBat-based harmonisation were evaluated across scanners.

**Results:**

After a median follow-up of 23 months, 29% of the patients recurred. No individual radiomic or clinical features were significantly associated with cancer recurrence. The best model was obtained using 10 TLR features combined with clinical information. The area under the curve (AUC), *F*_1_-score, precision and recall were respectively 0.78 (0.67–0.88), 0.49 (0.25–0.67), 0.42 (0.25–0.60) and 0.63 (0.20–0.80). ComBat did not improve the predictive performance of the best models. Both the TLR and the native models performance varied across scanners used in the test set.

**Conclusion:**

[^18^F]FDG PET radiomic features combined with ML add relevant information to the standard clinical parameters in terms of LACC patient’s outcome but remain subject to variability across PET/CT devices.

**Supplementary Information:**

The online version contains supplementary material available at 10.1007/s00259-021-05303-5.

## Introduction

Cervical cancer is the fourth most common cancer in women [1]. Currently, in clinical routine, the disease prognosis is based upon the FIGO/TNM staging system, with a particular emphasis on the lymph node involvement [2, 3]. Despite improved outcome, thanks to the introduction of concurrent chemoradiotherapy, the overall recurrence rate in patients with locally advanced cervical cancer (LACC) is 35%, and the median survival after recurrence is 10–12 months [2, 3]. Improving the patient risk stratification in order to adapt the treatment or surveillance schemes in high-risk patients would fulfil an unmet clinical need.

2-[^18^F]fluoro-2-deoxy-D-glucose ([^18^F]FDG) positron emission tomography combined with computed tomography (PET/CT) imaging plays an important role in treatment stratification in oncology. In cervical cancer, parameters such as the standard uptake value (SUV), metabolic tumour volume (MTV) or total lesion glycolysis (TLG) have been proposed as prognostic factors, although none has been integrated in the clinical decision algorithms [4]. Recently, there has been an increased interest in radiomics i.e. the characterisation of tumour phenotypes via the extraction of high-dimensional quantitative features from medical images, with the aim to support clinical decision-making [5–7]. Radiomic features have shown to predict treatment outcome in several cancer diseases including cervical cancer, and using various imaging modalities [8–11]. However, most of radiomic features show high sensitivity to multiple factors, including the scanner manufacturer and specific properties, acquisition protocols and the reconstruction algorithm and settings of each clinical center [12–18]. Radiomics have increasingly been combined with machine learning (ML) techniques in order to predict a specific clinical outcome [8, 10, 11, 19–26].

In this study, we first extracted radiomic features from multi-center/multi-scanner [^18^F]FDG PET images of cervical cancer and evaluated the performance of different classifiers combined with different feature selection (FS) methods to predict DFS. We hypothesised that a PET-based radiomics signature would have a significant prognostic value, higher or complementary to standard clinical parameters. We then evaluated the predictive value of tumour to liver ratios (TLR) of radiomic features [27]. The liver uptake is indeed quite homogeneous and reproducible [28], and we hypothesised that this may reduce the variability of uptake within the different patients and across centres. We also investigated the effect of several pre-processing steps of radiomics workflow applied before FS, including intensity discretisation scheme for textural features, the use of ComBat method for features harmonisation and image voxel size resampling, as well as the added value of clinical data and ComBat harmonisation after FS. Finally, we evaluated the performance of the trained models on data from each individual and external validation scanner, and we compared our radiomic signature with those previously developed by other research group [8].

## Materials and methods

### Patients and treatment information

One hundred and fifty-eight patients with LACC imaged between 2010 and 2016 were included in this retrospective study. All patients were treated with platinum-based chemotherapy and a combination of external radiotherapy (EBRT) (3D or not) and brachytherapy (BT), with a total dose of 85 Gy. Various regimens were applied depending on the treating Center: EBRT 45–50 Gy and pulse-dose rate brachytherapy (PDR) 35–40 Gy (*n* = 77); EBRT 45–50 Gy and high-dose brachytherapy (HDR) 4 × 6 or 7 Gy (*n* = 18); EBRT 60 Gy and PDR-BT 25 Gy (*n* = 1); EBRT 60 Gy and HDR-BT 3 × 7 Gy (*n* = 1); EBRT in addition to pulse-dose rate brachytherapy (PDR) with total dose of 60 to 70 Gy (*n* = 45). A complementary boost centered on the tumour was also given to 50 patients. A detailed description of patient clinical characteristics is given in Table 1. All patients had histologically proven cervical cancer and a median follow-up of 23 months (range: 4–84).

**Table 1:** Patient’s characteristics

	CHU Liège (Scanner A)	CHU Brest and ICO St Herbain (Scanner B)	Total	CHU Mcgill (Scanner C)
Number of patients	89	51	140	18
Age (median and range in years)	50 (23–76)	52 (23–82)	51 (23–82)	50 (28–86)
FIGO (%)				
IB1-IB2	18%	12%	16%	6%
IIA-IIB	66%	58%	64%	56%
IIIA-IIIB	12%	18%	14%	33%
IVA	3%	12%	6%	6%
Histology (% of SCC)				
	87%	82%	85%	89%
LN metastasis				
% of patients	19%	16%	18%	28%
Recurrence (%)	21%	35%	26%	50%

### PET/CT imaging

PET/CT studies were performed with 3 types of scanners. In the CHU of Liège, 89 studies were acquired using a Philips Gemini TF or BB (scanner A), and in the CHU of Brest and ICO St Herblain 17 and 34, respectively, were acquired using a Siemens Biograph mCT (scanner B). In addition, 18 studies performed with a General Electric Discovery ST (scanner C) at the McGill University Health Center were used as an external validation set. A mean activity of 306 MBq of [^18^F]FDG was injected before image acquisition with a mean uptake time of 66 min. The acquisition and reconstruction protocols are described in Table 1 of the supplementary data A.

### Radiomics workflow

The radiomics workflow usually consists of image acquisition or collection, image pre-processing (such as image interpolation, segmentation or intensity discretisation in the case of textures), extraction of the radiomic features and finally modelling [29]. We describe in the next sections the radiomics workflow used in this work.

### Images interpolation

PET images from scanners B and C, were interpolated in order to study the effect of image interpolation in the predictive performance of radiomics. We up-sampled or down-sampled the images using a linear method, so that all datasets had isotropic voxels of 4 mm^3^. Interpolation was done using a research toolbox (Oncoradiomics SA, Liège, Belgium).

### Segmentation

The 3D primary tumour volumes were segmented from the [^18^F]FDG PET images using the semi-automatic Fuzzy Local Adaptive Bayesian (FLAB) algorithm with 2 classes [30]. The median volume of the segmented lesions was 28.6 cm^3^ (range: 2.4–181.2 cm^3^). In addition, regions of 20 cm^3^ in the liver were manually drawn in order to investigate the predictive value of TLR radiomic features, as explained below. Those segmentations were reviewed and edited if needed by one nuclear medicine physician with 9 years of experience in clinical PET/CT. No volume cut-off value was applied for patient inclusion in this study.

### Radiomic features and intensity discretisation

Two hundred and fifteen features were extracted from the segmented volumes using the Oncoradiomics research toolbox. These features included first-order grey level statistics, geometry, fractals, texture matrix-based features and others. The detailed description of the features can be found in supplementary data B. For those standardised by the IBSI (Imaging biomarkers standardisation initiative), the implementation follows IBSI benchmark. We also studied the ratio of the features’ values calculated in the tumour and in the liver (TLR), except for the shape features. In addition to the radiomic features, data also included 4 clinical parameters i.e. FIGO stage (IB to IVA), histological types (squamous cell carcinoma (SCC) or not), age and presence of lymph node (LN) metastasis.

For the calculation of the texture matrix-based features, the intensities needed to be discretised. Image intensities were discretised using two different methods according to IBSI recommendations: fixed bin number (FBN, using 32 and 64 bins) and fixed bin width (FBW, with 4 different widths of 0.05, 0.1, 0.2 and 0.5 SUV) [29]. There was no missing data for any patient.

### ComBat harmonisation

ComBat is a batch adjustment method initially developed for genomics data [31, 32]. It has also been used in PET and CT studies to correct the center variability of radiomic features [33, 34]. In our work, we intended to use ComBat harmonisation to correct the effect of multi scanner acquisition and to test whether it could improve the predictive performance of predictive models based on radiomic features. First ComBat was applied with the non-parametric version, before FS. Since there was no significant difference in clinical characteristics of patients across centers, we did not use the covariate matrix modelling. Second, in a separate set of experiments, the best performance models were also retrained using ComBat harmonisation after FS.

### Statistical analysis

Clinical and treatment data from the different scanners were compared using chi square test for categorical data (FIGO, histology, presence of LN metastasis, type of EBRT i.e. 3D or not, complementary boost centered on the tumour and combination of EBRT/BT) to test the null hypothesis that the distribution of each of the parameters categories between the scanners were independent. For continuous data (age), we used a one-way ANOVA to test whether the difference in means differs or not. To predict DFS, a univariate Cox proportional hazard model was first applied to the clinical, treatment, radiomics and TLR radiomics data for each discretisation method. To this end, we separated our data into training and testing sets (80% and 20% of the data from each scanner, respectively). Training data was standardised with *z*-score normalisation before performing the Cox regression. The univariate Cox model was used to test the statistical significance (*P* value < = 0.05) of the features. Pearson correlation was computed between pairs of features. In case the correlation was higher than 0.9, the feature which was most correlated with all the others was removed. The Holm-Bonferroni correction method was used to correct for multiple hypothesis testing [35]. A multivariate Cox proportional hazard model was performed with the remaining features. Finally, the Youden index was used for extracting a threshold for the receiver operating characteristic (ROC) curve of the train data of each individual significant feature, clinical features and tumour volume (TV). Afterwards, the thresholds were used to plot Kaplan-Meier curves and evaluate individual features performance using recall, precision, and *F*_1_-score metrics. Differences in survival were evaluated using the log-rank test. In addition to the Cox model, we also evaluated whether combining different FS and ML classifiers methods was able to find a radiomics signature to predict DFS. For that purpose, we first dichotomised DFS into a binary endpoint i.e. recurrence or no recurrence, independently of the time of the recurrence. Next, we tested a different set of models, which differs in (i) the features type i.e. original radiomics (OR) or TLR radiomics, (ii) the pre-processing of the PET images i.e. with or without interpolation, (iii) the pre-processing of the features i.e. with or without ComBat harmonisation and intensity discretisation scheme, (iv) the FS and ML classifier method and (v) the metric used to optimise the number of features used in the model. Additionally, we also investigated the effect of adding clinical data before FS.

We tested 7 different FS methods: (1)- Accuracy decrease obtained from the embedded FS of the random forest (RF) classifier; (2)- Gini impurity decrease obtained from the embedded FS of the RF classifier; (3)- forward FS using maximum relevance minimum redundancy (MRMR) method with Pearson correlation; (4)- backward FS using MRMR with Pearson correlation; (5)- forward FS using MRMR with Spearman correlation; (6)- backward FS using MRMR with Spearman correlation and (7)- forward MRMR based on the mutual information (MI). We also used 4 ML classifiers: RF, support vector machine (SVM) with radial kernel, Naïve Bayes (NB) and a logistic regression (LR) [36–38]. The training data was used to tune the number of features selected by each FS method, which were limited to 10 in order to avoid overfitting. We used fivefold cross validation in our training data and chose the number of features to be used according to the best mean fold area under the curve (AUC) value, *F*-score (with Beta 1 and 2) and AUC of precision recall curve (AUCpr). Patients who recur were oversampled using the random oversampling method in the training data to help in the learning process [39]. We repeated this procedure for each of the models. We used for each classifier the default hyperparameters values in their respective R packages. Finally, for each of the different models with distinct selected features, all training data were bootstrapped with 1000 repetitions, in order to get confidence intervals for each performance metric, and tested on one test set independent of model/feature selection training set. We measured AUC, *F*_1_-score and *F*_2_-score, precision, recall, AUCpr and its corresponding percentile confidence intervals of the distinct models. We considered as best model the one with higher bootstrap *F*_1_-score values. A 0.5 probability threshold of the recurrence event was used to plot Kaplan-Meier curves. Survival curves were compared with the log-rank test. Delong test was used to compare AUC and a binomial test to compare precision of the best OR and TLR models [40].

The best performance models were retrained and retested adding clinical features and correcting multi-scanner variability using ComBat harmonisation after FS in order to investigate whether they could improve the models’ predictive ability. To decrease the overfitting risk of our models, we randomised the outcome of the test set and evaluate our model performance. By randomising the outcome, we expect to get an AUC close to 0.5.

**Fig. 1:**
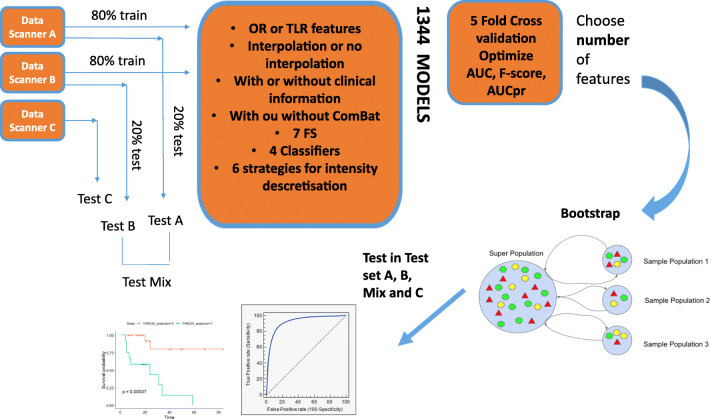
Radiomics pipeline

To test the dependency of prediction performance on data from different scanners, we replaced the test data belonging from all scanners with data from all the different scanners independently. We also validated our model in the data from scanner C, which was never seen in the training process. Statistical and ML analyses were performed using R software (Fig. 1).

Finally, we compared our models with the 3 statistical significant radiomic signatures (G1, G2 and G3) developed by Altazi et al. [8] for predicting local regional recurrence (LRR). We tested these radiomic signatures in the mix test set i.e. test set from scanner A + B, using the AUC. Predictor assessments were blinded for outcome or other predictors in all steps of this work.

## Results

After a median follow-up of 23 months, 29% of the patients recurred. There was no difference between the clinical characteristics of the 3 cohorts (*P* value: FIGO = 0.1084, presence of LN metastasis = 0.5302, histology = 0.7691 and age = 0.7205).

**Fig. 2:**
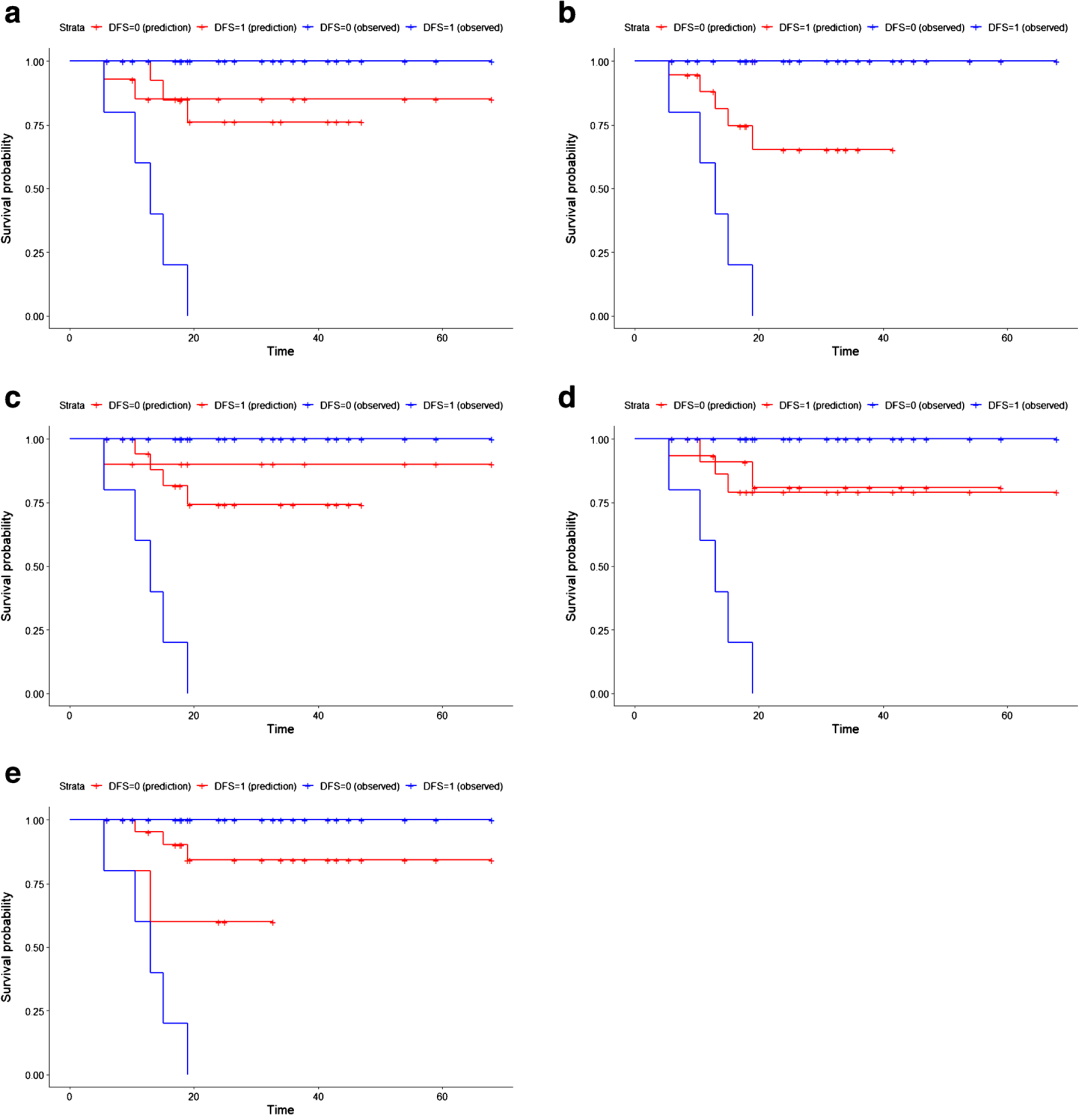
Kaplan-Meier curve of the each individual significant feature in univariate and multivariate analysis, after the Cox proportional hazard model. **a** GLDZM_DZNN_0.5 (OR) (Threshold = 0.59, log-rank test *P* value 0.079). **b** GLSZM_HILAE_0.5 (TLR) (Threshold = 0.07, log-rank test *P* value 0.044), **c** GLDZM_DZV_0.05 (TLR from interpolated images) (Threshold = 1.28, log-rank test *P* value 0.47). **d** Stats_qcod_0.2 (TLR from interpolated images) (Threshold = 1.18, log-rank test *P* value 0.088). **e** Histology (Threshold = 0.5, log-rank test *P* value 0.15)

With a cox proportional hazard model, clinical features, treatment scheme, TV, SUV_max_, MTV and TLG at any threshold, were not significantly associated with DFS in univariate analysis (Table 2). The significant features in the univariate and multivariate analyses are listed in Table 3. As features were standardised before performing the Cox regression, the hazard ratio units correspond to standard deviations of the different covariates. The most significant features were textural (GLDZM and GLSZM). Figure 2 shows the Kaplan-Meier curves for each of the significant individual features. The threshold for each feature was defined through the Youden Index of the feature ROC curve in the training data. GLSZM_HILAE_0.5 (TLR) was the only feature providing statistically different stratification between the two Kaplan-Meier curves, according to the log-rank test.

**Table 2:** Hazard ratios (HR) with 95% confidence intervals and *P* values of clinical features, as well as TV, MTV, SUV Max and TLG after performing a univariate Cox proportional hazard model to predict DFS. The Youden Index was used to find a threshold for each predictor, plot Kaplan-Meier curves and evaluate the performance of each individual feature in predicting DFS in the test set. AUC, recall, precision and *F*_1_-score were used as DFS performance metrics

*P* value KM curve	*F*_1_-score	Precision	Recall	AUC	*P* value in univariate analysis	HR (95% CI)	Feature
0.32	0.33	0.2	1	0.6	0.14	1.23 (0.94–1.6)	FIGO
0.15	0.4	0.4	0.4	0.64	0.33	1.14 (0.87–1.5)	Histology
0.67	0.22	0.25	0.2	0.54	0.15	1.22 (0.93–1.6)	Metastasis
0.027	0.44	0.5	0.4	0.61	0.84	0.97 (0.71–1.3)	Age
0.17	0	0	0	0.48	0.01	1.4 (1.1–1.9)	TV
0.23	0.36	0.24	0.8	0.48	0.65	1.06 (0.81–1.4)	MTV 50%
0.87	0.22	0.15	0.4	0.43	0.1	1.25 (0.96–1.6)	SUV MAX
0.67	0.15	0.13	0.2	0.43	0.32	1.14 (0.88–1.5)	TLG 50%

**Table 3:** Features which were significant in univariate and multivariate analysis. For each of the features, we show the hazard ratios (HR) with 95% confidence intervals and *P* values after performing the univariate Cox proportional hazard model. Additionally, we also show the AUC, recall, precision, *F*_1_-score and *P* value of KM curve. The threshold to measure the last metrics was defined using the Youden index of the training data set ROC curve. Feature’s names are described as in the supplementary data B with the additional information of the discretisation width and features origin (OR or TLR)

*P* value KM curve	*F*_1_-score	Precision	Recall	AUC	*P* value in multivariate analysis	*P* value in univariate analysis	HR (95% CI)	Feature
0.79	0.21	0.14	0.4	0.46	0.034	0.00049	0.44 (0.28–0.70)	GLDZM_DZNN_0.5 (OR)
0.044	0.43	0.28	0.1	0.68	0.0023	0.00058	1.61 (1.20–2.10)	GLSZM_HILAE_0.5 (TLR)
0.47	0.13	0.1	0.2	0.43	0.0016	0.001	1.65 (1.20–2.20)	GLDZM_DZV_0.05 (TLR from interpolated images)
0.088	0.24	0.14	0.8	0.61	0.0022	0.001	1.72 (1.20–2.40)	Stats_qcod_0.2 (TLR from interpolated images)
0.15	0.4	0.4	0.4	0.64	NA	0.002	0.65 (0.49–0.85)	Histology

The best results for predicting DFS were obtained with 10 TLR features i.e. 2 shape features: Shape_volume and Shape_centroidDistance; 3 texture matrix-based features: GLDZM_HIE, GLSZM_SZV and GLDZM_INN; 1 image intensity: Stats_var; and 4 intensity volume histogram features: IVH_RVRI_10, IVH_RVRI_20, IVH_AVRI_80 and IVH_AVRI_90) selected with the Forward MRMR MI, discretised with FBN (32 bins) and classified with RF. The AUC, *F*_1_-score 1, *F*_2_-score, precision, recall and AUCpr were 0.72 (0.62–0.8), 0.48 (0.25–0.67), 0.56 (0.22–0.74), 0.40 (0.22–0.60), 0.63 (0.20–0.80) and 0.50 (0.30–0.69), respectively. Adding all clinical data to the TLR features model further improved the results with AUC, *F*_1_-score, *F*_2_-score, precision, recall and AUCpr of 0.78 (0.67–0.88), 0.49 (0.25–0.67), 0.56 (0.22–0.74), 0.42 (0.25–0.60), 0.63 (0.2–0.80) and 0.53 (0.33–0.72).

Next were the 3 clinical features (age, histology, metastasis) combined with the following 6 radiomic features: one shape feature (Shape_elongation) and 5 intensity volume histogram features, (IVH_RVRI_50, IVH_RVRI_60, IVH_RVRI_70, IVH_RVRI_80 and IVH_RVRI_90); discretised using FBW (0.05 SUV), selected with the Backward MRMR Pearson method and a LR classifier. The AUC, *F*_1_-score, *F*_2_-score, precision, recall and AUCpr were 0.65 (0.47–0.73), 0.44 (0.25–0.57), 0.56 (0.32–0.69), 0.32 (0.18–0.44), 0.69 (0.40–0.80) and 0.38 (0.22–0.58), respectively. Adding the remaining clinical feature (FIGO) to the OR model did not improve prediction performances.

Neither using the ComBat-harmonized features nor applying ComBat harmonisation on these two models did improve performances (Supplementary data C).

**Fig. 3:**
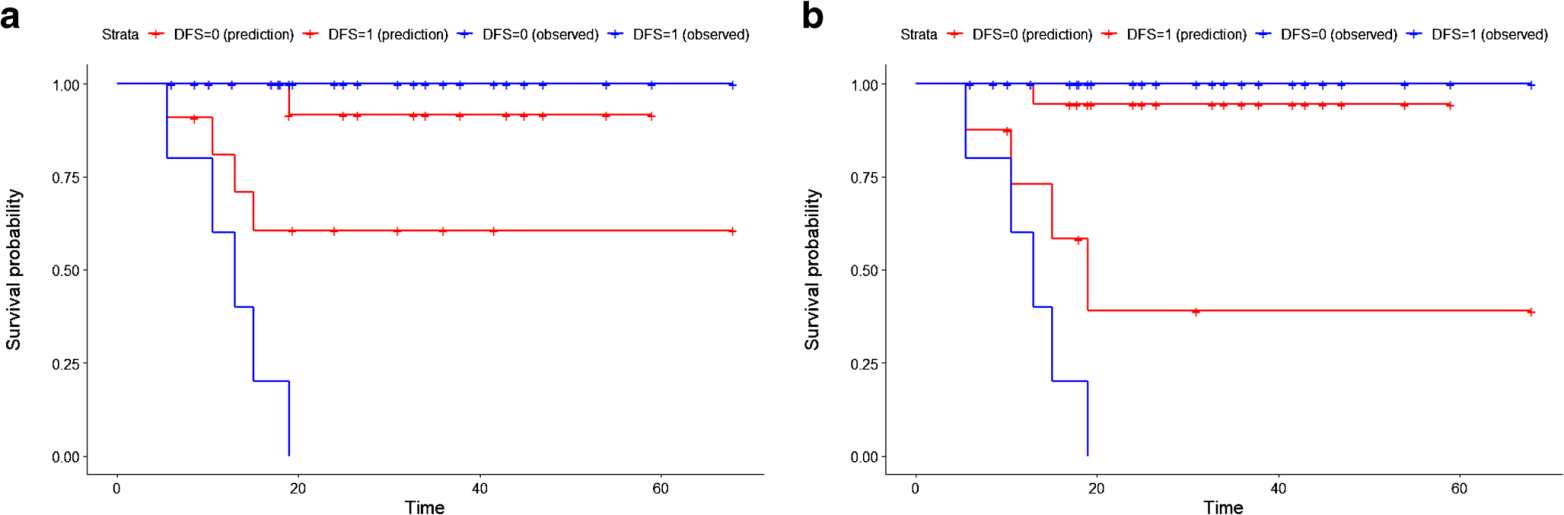
Kaplan-Meier curve of the test set for the best OR (**a**) and TLR (**b**) model. Red and blue curves represent respectively patients with better and worse prognosis. The log-rank test was used to estimate statistical significance of the difference between survival curves. The *P* value obtained from the log-rank test is shown in the left down corner of each image. The difference between both the Kaplan-Meier curves is statistical significant (log-rank *P* value = 0.034 for the OR model and 0.002 for the TLR model)

Figure 3 shows the Kaplan-Meier curves of the best OR and TLR models, which were significantly discriminant: log-rank *P* value of 0.034 for the OR and 0.002 for the TLR model. Both models performed better than the individual radiomic features. The differences between AUC and precision of the OR and TLR models were however not statistically significant (*P* value = 0.64 and 0.34 respectively).

By randomising the outcome of the test set, we obtain an AUC of 0.58 for the best OR model and an AUC of 0.56 in the best TLR model.

Training and testing in the independent scanners and on the external validation scanner resulted in large variations in the predictive ability of the models. Table 4 illustrates the variation in the distinct performance metrics for the best OR and TLR model.

**Table 4:** Variation of Bootstrap mean AUC, *F*_1_-score and *F*_2_-score, precision, recall and AUCpr values according to the different test data obtained from the different scanners. Table 4 (part a) shows the models using OR radiomics features and Table 4 (part b) those with TLR features. The 95% confidence intervals are in parentheses

	AUC	*F*_1_-score	*F*_2_-score	Precision	Recall	AUCpr
a						
Mix Scanner A and B	0.65 (0.47–0.73)	0.44 (0.25–0.57)	0.56 (0.32–0.69)	0.32 (0.18–0.44)	0.69 (0.40–0.80)	0.38 (0.22–0.58)
Scanner A	0.54 (0.24–0.67)	0.37 (0–0.57)	0.46 (0–0.63)	0.29 (0–0.5)	0.55 (0–0.67)	0.36 (0.15–0.72)
Scanner B	0.81 (0.50–1)	0.52 (0.25–0.67)	0.69 (0.36–0.83)	0.37 (0.17–0.50)	0.91 (0.50–1)	0.57 (0.27–1)
Scanner C	0.57 (0.36–0.75)	0.40 (0.14–0.67)	0.35 (0.12–0.61)	0.58 (0.20–1)	0.32 (0.11–0.56)	0.61 (0.46–0.82)
b						
Mix Scanner A and B	0.78 (0.67–0.88)	0.49 (0.25–0.67)	0.56 (0.22–0.74)	0.42 (0.25–0.60)	0.63 (0.2–0.8)	0.53 (0.33–0.72)
Scanner A	0.70 (0.56–0.84)	0.36 (0–0.57)	0.41 (0–0.63)	0.31 (0–0.50)	0.46 (0–0.67)	0.39 (0.25–0.65)
Scanner B	0.95 (0.78–1)	0.67 (0.4–1)	0.78 (0.45–1)	0.57 (0.29–1)	0.89 (0.50–1)	0.88 (0.58–1)
Scanner C	0.50 (0.37–0.65)	0.25 (0–0.46)	0.20 (0–0.38)	0.46 (0–0.75)	0.18 (0–0.33)	0.55 (0.46–0.67)

Finally, the radiomic signatures developed by Altazi et al. did not identify the patients with a higher risk of recurrence in our population, as shown in Table 5.

**Table 5:** Radiomic signatures developed by Altazi et al. The models were tested in the mix test set (test set A + B)

	AUC
Ref [8]	
G1	0.49
G2	0.56
G3	0.6

## Discussion

There are conflicting results regarding the prognostic value of [^18^F]FDG uptake in cervical cancer when using conventional metrics such as the SUV_max_, MTV or TLG [8, 9, 41–44]. We recently found, in a different patient’s sample, TLG to be the only parameter associated with DFS in multivariate analysis [45]. In the current multicenter series however, except histology, none of the clinical or conventional metabolic features such as SUV, MTV and TLG were statistically significant predictors of DFS in univariate analysis. Compared to De Cuypere et al., the follow-up is slightly shorter in the present study, but the population size is larger, which consequently may be statistically more relevant. Moreover, the tumours were segmented using different methods in the two studies, which is a factor known to affect the TLG and also radiomic features reproducibility [46]. Of note, even though the radiation therapy protocols varied across centers, this did not intervene in the DFS. Radiomics has been proposed for characterising cervical cancer subtypes [47] or predicting the response to treatment either based upon [^18^F]FDG PET/CT alone [8, 11, 48] or in combination with MRI [9] [49, 50]. In the current series, we confirm that individual radiomic features, in particular matrix-based and intensity histogram, are significant predictors of DFS in uni- and multivariate analysis. However, the best overall performances are obtained with a ML model that uses 10 TLR radiomic features characterising different tumour properties such as shape, texture and intensity. The best model was obtained when features were selected using a forward MRMR MI method and classified with a RF. The good performance of this classifier has been already observed by other studies [20–22]. Furthermore, we found combining clinical and radiomic features improve the prediction ability of the models. Regarding the intensity discretisation scheme, the application of the FBW discretisation method in PET has been recommended [15, 29], although some studies have also reported more favourable properties using FBN [51]. This is related to the fact that FBN and FBW have different drawbacks and advantages. FBW preserves the relationship between PET units and the corresponding physical meaning, contrary to arbitrary units (such as in some non-quantitative MRI sequences). However, most features cannot be directly compared across different volumes of interest. FBN on the other hand does not preserve the relationship between intensities and physical meaning but introduces a normalisation effect that can be favourable when contrast is considered important. Furthermore, it allows direct comparison of values across different volumes of interest. In our findings, the best model was obtained when using a FBN (32 bins) discretisation method.

We also looked at various parameters known to affect the reproducibility and robustness of such process. Feature selection in particular is an important step of the analysis process as it improves the generalisation of the results. It is known that each FS method and each classifier has its own limitations, and indeed, like other researchers in various settings, we did observe a large performance variability when using different methods for discretisation, FS and classification [14–17, 19–22]. In addition, we also evaluated the effect of image interpolation on the model performances. In radiomics studies, it is recommended for most textural features to ensure isotropic voxel sizes across observations or patients. Isotropic voxel sizes make textural features rotationally invariant, allows direct comparison between the different samples and improves features reproducibility [29]. However, different methods for interpolating images voxels exists, which result in distinct textural feature values [17] and predictive model performances [52]. In addition to this, image interpolation also implies information inference or loss, depending on whether down-sampling or up-sampling of the voxel size is performed. In our study, the best radiomics model was obtained when interpolating images into an isotropic voxel size of 4 × 4 × 4 mm^3^.

Multi-center radiomics studies imply features variability mainly due to distinct imaging devices and protocols. This variability is among the major obstacles preventing generalisation of radiomics signatures in the clinical practice. Different methods have been developed to decrease radiomic features variability across clinical centers. Harmonising acquisition and reconstruction parameters, for instance through the EANM EARL initiative might contribute reaching such goal. A key finding of the present study is that the TLR perform better across scanners than the native radiomics features. As each patient acts as his own control, it should not come as a surprise finding the ratios as more stable than the native features. ComBat is another method that was previously successfully used for that purpose. It was shown to align features distributions across different clinical centers as well as to improve predictive ability of radiomic features namely in cervical cancer DFS prediction [50]. In our dataset, the best model was not obtained when applying ComBat harmonisation before FS. Additionally, ComBat did not improve model performance when applied after FS in the best OR and TLR models. As observed here, ComBat might therefore not be unequivocally effective, as it presents drawbacks such as the assumption that the site effect follows a Gaussian and Inverse-Gama distribution and the need for sample sizes large enough to be statistically representative. Studies have shown in MRI that ComBat might not preserve biological variability, and minor differences in pre-processing steps can lead to unexpected effects during ComBat harmonizsation [53, 54].

There is currently no satisfying answer to the only clinically relevant question when it comes to introducing ML and radiomics in the clinical field of nuclear medicine: Is there a “universal” feature set or ML strategy that could predict the outcome, in this case DFS in cervical cancer patients, independently of the PET/CT device and imaging methodology?. Indeed, in lung cancer for instance, Parmar et al. [21] and Sun et al. [19] compared different FS methods and classifiers and reached very different conclusions. Deist et al. [22] also observed that when training the model with different datasets, the performance of classifiers is significantly different. We similarly observed that the performance of our models is highly dependent on the data used to train and test the models, which can be related to the relatively small sizes of the datasets, compared to other ML applications where thousands or tens of thousands of samples are available for training the models. We also found that the radiomic features or models selected and developed by other researchers were not transposable to our population i.e. could not predict recurrence in our patients, with the caveat that the previous works did not evaluate DFS, but rather LRR [8]. In particular, none of the parameters selected in the models developed by Altazi et al. [8] were common to our model, except GLSZM_SZV (size zone variance from GLSZM matrix, a texture feature) when used as a ratio with the liver. In Lucia et al. [9] GLNU_GLRLM was the only PET feature predictive of DFS, and this parameter was not selected in our models. Interestingly, this parameter was identified as significant in Lucia et al. [9], but only when calculated following discretisation with histogram equalisation, not after FBW or FBN discretisation. We did not include discretisation with histograms, as it is currently not included in the IBSI standard. This illustrates that the devil might indeed be in the details and further emphasises the need for harmonised feature generation and robust models across centers and scanners.

Our study presents some limitations. The tumour segmentations were done by a single observer, using the semi-automatic FLAB algorithm. Using fully automatic and thus possibly more reproducible segmentation methods [48] [55] might improve the results, although considering that these tumours are consistently highly hypermetabolic and easily delineated, we do not expect major changes. Additionally, it might also be relevant to evaluate features not only on the segmented region but also for instance in regions around the tumour, as proposed by Hao et al. [11]. Finally, in this study, we dichotomised DFS into a binary outcome and predicted DFS without considering time-to-event information. Leger et al. [20] reported that this simplification can bias the models. However, we believe that this is not clinically relevant since patients that have a recurrence within a relatively short time, which is the case in our data, are treated equally. Our results show that combining distinct radiomics and clinical information can help in the stratification of patients with high and low risks of recurrence. However, the predictive performances of the metabolic data do not appear good enough to be applied in clinical practice and need further validation in larger multi-center cohorts of patients. Further improvements could nonetheless be obtained by combining radiomic features extracted from other image modalities, such as MRI, as shown by Lucia et al. [9].

Despite these limitations, to the best of our knowledge, this is the first study to investigate TLR radiomic features in predicting DFS in cervical cancer. All the features calculations were performed using a software which is documented and fully in line with IBSI standardisation, which should help other research groups reproduce our results. Our study follows the TRIPOD guidelines [56] (Supplementary data D) and scores 42% according to the radiomics quality score, which compares favourably with the majority of previous radiomics studies [7]. We believe this is a step towards a possible integration of radiomics in the clinics, which is slow to come and hampered by methodological drawbacks, as illustrated by recent articles [57–59].

Future research should include combining different image modalities [9] [23] and combining features discretised with different schemes instead of considering each discretisation set independently. The former is subject to its own inter-device variability limitations and the latter is more demanding in terms of computation time. An exhaustive hyper parameter tuning of ML models could also be investigated for evaluating the improvement of performance. Fusion or combination of the different FS and ML techniques could also help in alleviating the variability of resulting models and increase performance.

## Conclusion

In this multicentre series, a ML algorithm with 10 TLR radiomic features combined with the clinical information resulted in the best predictor of cancer recurrence. Despite these encouraging results, one cannot ignore the persistence of significant dependency of the features on the local acquisition settings as well as the poor F-score performance. Hence, further works need to be done prior to large-scale clinical testing.

## Supplementary Information

ESM 1 A) Patients [^18^F] FDG PET protocol description according to Scanner. (PDF 363 kb)

ESM 2 B) Oncoradiomics features description (PDF 585 kb)

ESM 3 C) Bootstrap mean AUC, *F*_1_-score and *F*_2_-score, precision, recall and AUCpr values of the best models using ComBat harmonization (PDF 125 kb)

ESM 4 D) TRIPOD adherence data extraction checklist (PDF 871 kb)

## Data Availability

https://github.com/msilvaferreira/Phd/tree/master/FDG%20PET%20radiomics%20to%20predict%20disease%20free%20survival%20in%20Cervical%20Cancer.
