# Correction to: [^18^F]FDG PET radiomics to predict disease-free survival in cervical cancer: a multi-scanner/center study with external validation

**DOI:** 10.1007/s00259-021-05397-x

**Published:** 2021-05-26

**Authors:** Marta Ferreira, Pierre Lovinfosse, Johanne Hermesse, Marjolein Decuypere, Caroline Rousseau, François Lucia, Ulrike Schick, Caroline Reinhold, Philippe Robin, Mathieu Hatt, Dimitris Visvikis, Claire Bernard, Ralph T. H. Leijenaar, Frédéric Kridelka, Philippe Lambin, Patrick E. Meyer, Roland Hustinx

**Affiliations:** 1 GIGA-CRC in vivo Imaging, University of Liège, GIGA, Avenue de l’Hôpital 11, 4000 Liege, Belgium; 2 Division of Nuclear Medicine and Oncological Imaging, University Hospital of Liège, Liège, Belgium; 3 Department of Radiation Oncology, Liège University Hospital, Liège, Belgium; 4 Division of Oncological Gynecology, University Hospital of Liège, Liège, Belgium; 5 Université de Nantes, CNRS, Inserm, CRCINA, F-44000 Nantes, France; 6 ICO René Gauducheau, F-44800 Saint-Herblain, France; 7 Radiation Oncology Department, University Hospital, Brest, France; 8 LaTIM, INSERM, UMR 1101, Univ Brest, Brest, France; 9 Department of Radiology, McGill University Health Centre (MUHC), Montreal, Canada; 10 Department of Nuclear Medicine and EA3878, Brest University Hospital, University of Brest, Brest, France; 11 Oncoradiomics SA, Clos Chanmurly 13, 4000 Liège, Belgium; 12 The-D Lab, Precision Medicine, GROW-School for Oncology and Developmental Biology, Maastricht University Medical Centre, Maastricht, Netherlands; 13 Department of Radiology and Nuclear Medicine, Maastricht University Medical Centre, Maastricht, The Netherlands; 14 Bioinformatics and Systems Biology Lab, University of Liège, Liège, Belgium

**Correction to Eur J Nucl Med Mol Imaging**

10.1007/s00259-021-05303-5

The article “[^18^F]FDG PET radiomics to predict disease-free survival in cervical cancer: a multi-scanner/center study with external validation”, written by Marta Ferreira, Pierre Lovinfosse, Johanne Hermesse, Marjolein Decuypere, Caroline Rousseau, François Lucia, Ulrike Schick, Caroline Reinhold, Philippe Robin, Mathieu Hatt, Dimitris Visvikis, Claire Bernard, Ralph T. H. Leijenaar, Frédéric Kridelka, Philippe Lambin, Patrick E. Meyer, and Roland Hustinx, was originally published Online First without Open Access. After being published online, the authors decided to opt for Open Choice and to make the article an Open Access publication. Therefore, the copyright of the article has been changed to © The Author(s) 2021 and the article is forthwith distributed under the terms of the Creative Commons Attribution 4.0 International License, which permits use, sharing, adaptation, distribution and reproduction in any medium or format, as long as you give appropriate credit to the original author(s) and the source, provide a link to the Creative Commons licence, and indicate if changes were made. The images or other third party material in this article are included in the article's Creative Commons licence, unless indicated otherwise in a credit line to the material. If material is not included in the article's Creative Commons licence and your intended use is not permitted by statutory regulation or exceeds the permitted use, you will need to obtain permission directly from the copyright holder. To view a copy of this licence, visit http://creativecommons.org/licenses/by/4.0/.

