# Supplementary material for: [18F]FDG PET radiomics to predict disease-free survival in cervical cancer: a multi-scanner/center study with external validation
Source: Eur J Nucl Med Mol Imaging. 2021 Mar 26;48(11):3432–43. doi: 10.1007/s00259-021-05303-5 (PMC8440288; doi:10.1007/s00259-021-05303-5)
Supplement: Supplementary file 1 — A) Patients [18F] FDG PET protocol description according to Scanner. (PDF 363 kb) [file 259_2021_5303_MOESM1_ESM.pdf]

Supplementary data A

Table 1

Patients [^{18}F] FDG PET protocol description according to Scanner.

	Number of Patients	*Mean uptake time (min)*	*Mean injected activity and range (Mbq)*	*Voxel size (mm^3)*	*Reconstruction method*
Scanner A	89	67	227 (120-461)	64	BLOB-OS-TF
Scanner B	51	59	214 (104-399)	32	PSF+TOF 2i21s
Scanner C	18	68	434 (268-497)	75	VPFXS, OSEM or 3D IR
