# Supplementary material for: [18F]FDG PET radiomics to predict disease-free survival in cervical cancer: a multi-scanner/center study with external validation
Source: Eur J Nucl Med Mol Imaging. 2021 Mar 26;48(11):3432–43. doi: 10.1007/s00259-021-05303-5 (PMC8440288; doi:10.1007/s00259-021-05303-5)
Supplement: Supplementary file 2 — B) Oncoradiomics features description (PDF 585 kb) [file 259_2021_5303_MOESM2_ESM.pdf]

RadiomiX

Feature descriptions

Version 20190207

©2019 OncoRadiomics

Table of contents

Table of contents 2
Definition of RadiomiX features 3
First-order gray-level statistics 3
Fractal Dimension features 6
Geometric features 7
Gray-Level Co-Occurrence Matrix based features 10
Gray-Level Run-Length matrix based features 15
Gray-Level size-zone matrix based features 18
Gray-Level distance-zone matrix based features 21
Intensity histogram features 24
Intensity volume histogram (IVH) features (PET images only) 29
Local Intensity features 30
Neighborhood gray tone difference matrix based features 31
Neighboring gray level dependence matrix based features 32
Radial gradient and radial deviation features (CT images only) 36
Filtered features 38
Wavelet features 38
Laplacian of Gaussian features 40
References 41

Definition of RadiomiX features

First-order gray-level statistics

First-order gray-level statistics describe the distribution of gray-values within the volume. Let X denote the three dimensional image matrix with N voxels, P the first order histogram, $P(i)$ the fraction of voxels with intensity level i and N_l the number of discrete intensity levels.

1. Energy

$$energy = \sum_{i=1}^{N} X(i)^2$$

Energy is also known as the sum of squares.

2. Entropy

$$entropy = \sum_{i=1}^{N_l} P(i) \log_2 P(i)$$

3. Kurtosis

$$kurtosis = \frac{\frac{1}{N}\sum_{i=1}^{N}(X(i) - \bar{X})^4}{\left(\frac{1}{N}\sum_{i=1}^{N}(X(i) - \bar{X})^2\right)^2}$$

where $\bar{X}$ is the mean of X.

4. Maximum

The maximum intensity value of X.

$$maximum = \max(X)$$

5. Mean

The mean gray-value of X.

$$mean = \frac{1}{N}\sum_{i=1}^{N} X(i)$$

6. Mean absolute deviation

The mean of the absolute deviations of all voxel intensities around the mean intensity value.

$$mean\ absolute\ deviation = \frac{1}{N}\sum_{i=1}^{N}\left|X(i) - \bar{X}\right|$$

where $\bar{X}$ is the mean of X.

7. Median

The sample median of X, or the 50th percentile of X.

8. Minimum

The minimum intensity value of X.

$$minimum = \min(X)$$

9. Range

The range of intensity values of X.

$$range = \max(X) - \min(X)$$

10. Root mean square (RMS)

The quadratic mean, or the square root of the mean of squares of all voxel intensities.

$$RMS = \sqrt{\frac{\sum_{i}^{N} X(i)^2}{N}}$$

11. Skewness

$$skewness = \frac{\frac{1}{N}\sum_{i=1}^{N}(X(i) - \bar{X})^3}{\left(\sqrt{\frac{1}{N}\sum_{i=1}^{N}(X(i) - \bar{X})^2}\right)^3}$$

where $\bar{X}$ is the mean of X.

12. Standard deviation

$$standard\ deviation = \left(\frac{1}{N-1}\sum_{i=1}^{N}(X(i) - \bar{X})^2\right)^{1/2}$$

where $\bar{X}$ is the mean of X.

13. Robust mean absolute deviation

The mean absolute deviation (0) of only those voxels in X with a gray-value between the 10th and 90th percentile.

14. 10th percentile

The 10th percentile of X, a robust alternative to the minimum gray-value (8).

15. 90th percentile

The 90th percentile of X, a robust alternative to the maximum gray-value (4).

16. Interquartile range

The interquartile range is defined as the 75th minus the 25th percentile of X.

17. Uniformity

$$uniformity = \sum_{i=1}^{N_l} P(i)^2$$

18. Variance

$$variance = \frac{1}{N-1} \sum_{i=1}^{N} (X(i) - \bar{X})^2$$

where $\bar{X}$ is the mean of X. Variance is the square of the standard deviation (12).

Fractal Dimension features

The Fractal Dimension (FD) of the image is computed as described by Al-Kadi and Watson (1). Given the FD processed image I, with N elements:

19. Average:

$$average = \frac{1}{N}\sum I$$

20. Lacunarity

$$lacunarity = \frac{\frac{1}{N}\sum I^2}{\frac{1}{N^2}\sum I} - 1$$

21. Standard deviation:

$$standard\ deviation = \left(\frac{1}{N-1}\sum (I - \bar{I})^2\right)^{1/2}$$

Where $\bar{I}$ is the mean of I.

Geometric features

Geometric features describe the shape and size of the volume of interest. Let V be the volume and A the surface area of the volume of interest. Let N be the total number of voxels, $X = \{\vec{X}_1, \vec{X}_2, \dots, \vec{X}_N\}$ the set of N Cartesian coordinate vectors and $I = \{I_1, I_2, \dots, I_N\}$ the corresponding intensity values.

22. Asphericity

$$asphericity = \left(\frac{1}{36\pi}\frac{A^3}{V^2}\right)^{\frac{1}{3}} - 1$$

23. Centroid distance

The centroid distance is the Euclidean distance between the geometric centroid (C_g) and the centroid weighing each voxel by its intensity value (C_i). The centroid distance is a measure of how close the high intensity values are to the geometric center.

$$C_g = \frac{1}{N}\sum_{i=1}^{N} \vec{X}_i$$

$$C_i = \frac{\sum_{i=1}^{N} I\vec{X}_i}{\sum_{i=1}^{N} I}$$

$$centroid\ distance = \left\|C_g - C_i\right\|$$

24. Compactness 1

Compactness is a measure of how much the volume resembles a sphere, as described by Aerts et al. (2).

$$compactness\ 1 = \frac{V}{\sqrt{\pi}A^{\frac{2}{3}}}$$

25. Compactness 2

$$compactness\ 2 = 36\pi\frac{V^2}{A^3}$$

26. Compactness 3

$$compactness\ 3 = \frac{V}{\sqrt{\pi}A^{\frac{3}{2}}}$$

A dimensionless alternative to Compactness (24), as described by Aerts et al. (2)

27. Maximum diameter

The maximum diameter is the largest pairwise difference between voxels on the surface of the volume, in 3D and for each plane separately. The following diameters are calculated:

27.1. The maximum three-dimensional tumor diameter.

27.2. The maximum two-dimensional diameter of all transversal planes.

27.3. The maximum two-dimensional diameter of all sagittal planes.

27.4. The maximum two-dimensional diameter of all coronal planes.

28. Major axis length

Axis lengths are measures of the extent of the volume along its three principle axis. Principle component analysis (PCA) on the x, y and z coordinates of all voxels within the volume is used to determine the three orthogonal eigenvectors and corresponding eigenvalues ($\lambda_{max}, \lambda_{minor}, \lambda_{min}$). The major axis length is the largest eigenvalue (λ_{max}) as determined by PCA.

29. Minor axis length

The largest eigenvalue (λ_{minor}) as determined by PCA.

30. Least axis length

The smallest eigenvalue (λ_{min}) as determined by PCA.

31. Elongation

$$elongation = \frac{\lambda_{minor}}{\lambda_{max}}$$

32. Flatness

$$flatness = \frac{\lambda_{min}}{\lambda_{max}}$$

33. Spherical disproportion (3)

Spherical disproportion is a measure of how much the volume resembles a sphere.

$$spherical\ disproportion = \frac{A}{4\pi R^2}$$

Where A is the surface area and R is the radius of a sphere with the same volume as the tumor, obtained through:

$$R = \sqrt[3]{\frac{3V}{4\pi}}$$

34. Sphericity (3)

Sphericity is a measure of how much the volume resembles a sphere.

$$sphericity = \frac{\pi^{\frac{1}{3}}(6V)^{\frac{2}{3}}}{A} = \frac{(36\pi V^2)^{\frac{1}{3}}}{A}$$

35. Surface area

The surface area is calculated by triangulation (i.e. dividing the surface into connected triangles, which define the isosurface enclosing the volume) and is defined as:

$$surface\ area = \sum_{i=1}^{N} \frac{1}{2} |a_i b_i \times a_i c_i|$$

Where N is the total number of triangles covering the surface and a, b and c are edge vectors of the triangles.

36. Surface to volume ratio

$$surface\ to\ volume\ ratio = \frac{A}{V}$$

37. Volume

The volume is defined as the number of voxels within the volume multiplied by the voxel volume.

$$volume = Nv$$

Where v is the volume of a single voxel.

Gray-Level Co-Occurrence Matrix based features

Gray level co-occurrence matrix (GLCM) based features, as originally described by Haralick et al (4). A normalized GLCM is defined as $P(i,j;\delta,\alpha)$, a matrix with size $N_g \times N_g$ describing the second-order joint probability function of an image, where the (i,j)th element represents the number of times the combination of intensity levels i and j occur in two pixels in the image, that are separated by a distance of δ pixels in direction α, and N_g is the maximum discrete intensity level in the image. Let:

$P(i,j)$ be the normalized (i.e. $\sum P(i,j) = 1$) co-occurrence matrix, generalized for any δ and α ,

$p_x(i) = \sum_{j=1}^{N_g} P(i,j)$,

$p_y(j) = \sum_{i=1}^{N_g} P(i,j)$,

μ_x be the mean of p_x, where $\mu_x = \sum_{i=1}^{N_g} \sum_{j=1}^{N_g} iP(i,j)$

μ_y be the mean of p_y, where $\mu_y = \sum_{i=1}^{N_g} \sum_{j=1}^{N_g} jP(i,j)$

σ_x be the standard deviation of p_x, where $\sigma_x^2 = \sum_{i=1}^{N_g} \sum_{j=1}^{N_g} P(i,j)(i-\mu_x)^2$

σ_y be the standard deviation of p_y, where $\sigma_y^2 = \sum_{i=1}^{N_g} \sum_{j=1}^{N_g} P(i,j)(j-\mu_y)^2$

$p_{x+y}(k) = \sum_{i=1}^{N_g} \sum_{j=1}^{N_g} P(i,j),\ i+j=k,\ k=2,3,\dots,2N_g$,

$p_{x-y}(k) = \sum_{i=1}^{N_g} \sum_{j=1}^{N_g} P(i,j),\ |i-j|=k,\ k=0,1,\dots,N_g-1$,

$HXY1 = -\sum_{i=1}^{N_g} \sum_{j=1}^{N_g} P(i,j) \ln(p_x(i)p_y(j))$,

$HXY2 = -\sum_{i=1}^{N_g} \sum_{j=1}^{N_g} p_x(i)p_y(j) \ln(p_x(i)p_y(j))$,

$HX = -\sum p_x \ln(p_x)$

$HY = -\sum p_y \ln(p_y)$

38. Average (μ)

$$average\ (\mu) = \frac{\sum_{i=1}^{N_g} \sum_{j=1}^{N_g} (i+j)P(i,j)}{2}$$

Note that for a symmetrical GLCM, $\mu = \mu_x = \mu_y$.

39. **Autocorrelation**

$$autocorrelation = \sum_{i=1}^{N_g} \sum_{j=1}^{N_g} ijP(i,j)$$

40. **Cluster Prominence**

$$cluster\ prominence = \sum_{i=1}^{N_g} \sum_{j=1}^{N_g} \left[i + j - \mu_x - \mu_y\right]^4 P(i,j)$$

41. **Cluster Shade**

$$cluster\ shade = \sum_{i=1}^{N_g} \sum_{j=1}^{N_g} \left[i + j - \mu_x - \mu_y\right]^3 P(i,j)$$

42. **Cluster Tendency**

$$cluster\ tendency = \sum_{i=1}^{N_g} \sum_{j=1}^{N_g} \left[i + j - \mu_x - \mu_y\right]^2 P(i,j)$$

43. **Contrast (5)**

$$contrast = \sum_{i=1}^{N_g} \sum_{j=1}^{N_g} |i - j|^2 P(i,j) = \sum_{k=0}^{N_g - 1} k^2\, p_{x-y}(k)$$

44. **Correlation**

$$correlation = \frac{\sum_{i=1}^{N_g} \sum_{j=1}^{N_g} ijP(i,j) - \mu_x \mu_y}{\sigma_x \sigma_y}$$

45. **Difference Average (μ_{x-y})**

$$difference\ average\ (\mu_{x-y}) = \sum_{k=0}^{N_g - 1} kp_{x-y}$$

46. **Difference Entropy**

$$difference\ entropy = -\sum_{i=0}^{N_g - 1} P_{x-y}(i) \log_2\left[P_{x-y}(i)\right]$$

47. Difference Variance

$$difference\ variance = \sum_{i=0}^{N_g-1} (i - \mu_{x-y})^2 P_{x-y}(i)$$

48. Dissimilarity

$$dissimilarity = \sum_{i=1}^{N_g} \sum_{j=1}^{N_g} |i-j| P(i,j)$$

49. Energy (6)

$$energy = \sum_{i=1}^{N_g} \sum_{j=1}^{N_g} [P(i,j)]^2$$

This feature is also called Angular Second Moment (ASM) and Uniformity (5).

50. Entropy (H)

$$entropy\ (H) = -\sum_{i=1}^{N_g} \sum_{j=1}^{N_g} P(i,j) \log_2[P(i,j)]$$

51. Homogeneity 1

$$homogeneity\ 1 = \sum_{i=1}^{N_g} \sum_{j=1}^{N_g} \frac{P(i,j)}{1 + |i-j|}$$

This feature is also called Inverse Difference (5).

52. Homogeneity 2 (6)

$$homogeneity\ 2 = \sum_{i=1}^{N_g} \sum_{j=1}^{N_g} \frac{P(i,j)}{1 + |i-j|^2}$$

This feature is also called Inverse Difference Moment (5).

53. Informational measure of correlation 1 (IMC1)

$$IMC1 = \frac{H - HXY1}{\max\{HX, HY\}}$$

Where H is the entropy (50).

54. Informational measure of correlation 2 (IMC2)

$$IMC2 = \sqrt{1 - e^{-2(HXY2 - H)}}$$

Where H is the entropy (50).

55. Inverse Difference Moment Normalized (IDMN)

$$IDMN = \sum_{i=1}^{N_g} \sum_{j=1}^{N_g} \frac{P(i,j)}{1 + \left(\frac{|i-j|^2}{N_g^2}\right)}$$

56. Inverse Difference Normalized (IDN)

$$IDN = \sum_{i=1}^{N_g} \sum_{j=1}^{N_g} \frac{P(i,j)}{1 + \left(\frac{|i-j|}{N_g}\right)}$$

57. Inverse variance

$$inverse\ variance = \sum_{i=1}^{N_g} \sum_{j=1}^{N_g} \frac{P(i,j)}{|i-j|^2} \quad , i \neq j$$

58. Maximal Correlation Coefficient

$$maximal\ correlation\ coefficient = \sqrt{\text{second largest eigenvalue of } Q}$$

$$Q = \sum_{k=1}^{N_g} \frac{P(i,k)P(j,k)}{p_x(i)p_y(k)}$$

59. Maximum Probability

$$maximum\ probability = \max\{P(i,j)\}$$

60. Sum average (SA)

$$sum\ average\ (SA) = \sum_{i=2}^{2N_g} \left[iP_{x+y}(i)\right]$$

61. Sum entropy

$$sum\ entropy = -\sum_{i=2}^{2N_g} P_{x+y}(i) \log_2\left[P_{x+y}(i)\right]$$

62. Sum variance

$$sum\ variance = \sum_{i=2}^{2N_g} (i - SA)^2 P_{x+y}(i)$$

63. Variance (sum of squares)

$$variance = \sum_{i=1}^{N_g} \sum_{j=1}^{N_g} (i - \mu)^2 P(i,j)$$

Gray-Level Run-Length matrix based features

Gray-level run-length matrix (GLRLM) based features, as described by Galloway et al. (7). Run length metrics quantify gray level runs in an image. A gray level run is defined as the length in number of pixels, of consecutive pixels that have the same gray level value. In a gray level run length matrix $p(i,j|\theta)$, the (i,j)th element describes the number of times j a gray level i appears consecutively in the direction specified by θ. Let:

$p(i,j)$ be the (i,j)th entry in the given run-length matrix p, generalized for any direction θ,

N_g the number of discrete intensity values in the image,

N_r the maximum run length,

N_s the total number of runs, where $N_s = \sum_{i=1}^{N_g} \sum_{j=1}^{N_r} p(i,j)$,

p_r the sum distribution of the number of runs with run length j, where $p_r(j) = \sum_{i=1}^{N_g} p(i,j)$,

p_g the sum distribution of the number of runs with gray level i, where $p_g(i) = \sum_{j=1}^{N_r} p(i,j)$,

N_p the number of voxels in the image, where $N_p = \sum_{j=1}^{N_r} j p_r$,

$p_n(i,j)$ the normalized run-length matrix, where $p_n(i,j) = \frac{p(i,j)}{N_s}$,

μ_r the mean run length, where $\mu_r = \sum_{i=1}^{N_g} \sum_{j=1}^{N_r} j p_n(i,j)$,

μ_g the mean gray level, where $\mu_g = \sum_{i=1}^{N_g} \sum_{j=1}^{N_r} i p_n(i,j)$.

64. Short Run Emphasis (SRE)

$$SRE = \frac{1}{N_s} \sum_{j=1}^{N_r} \frac{p_r}{j^2}$$

65. Long Run Emphasis (LRE)

$$LRE = \frac{1}{N_s} \sum_{j=1}^{N_r} j^2 p_r$$

66. Gray Level Non-Uniformity (GLN)

$$GLN = \frac{1}{N_s} \sum_{i=1}^{N_g} p_g^2$$

67. **Gray Level Non-Uniformity Normalized (GLNN)**

$$GLNN = \frac{1}{N_s^2}\sum_{i=1}^{N_g} p_g^2$$

68. **Run Length Non-Uniformity (RLN)**

$$RLN = \frac{1}{N_s}\sum_{j=1}^{N_r} p_r^2$$

69. **Run Length Non-Uniformity Normalized (RLNN)**

$$RLNN = \frac{1}{N_s^2}\sum_{j=1}^{N_r} p_r^2$$

70. **Run Percentage (RP)**

$$RP = \frac{N_s}{N_p}$$

71. **Low Gray Level Run Emphasis (LGRE)**

$$LGRE = \frac{1}{N_s}\sum_{i=1}^{N_g} \frac{p_g}{i^2}$$

72. **High Gray Level Run Emphasis (HGRE)**

$$HGRE = \frac{1}{N_s}\sum_{i=1}^{N_g} i^2 p_g$$

73. **Short Run Low Gray Level Emphasis (SRLGE)**

$$SRLGE = \frac{1}{N_s}\sum_{i=1}^{N_g}\sum_{j=1}^{N_r} \frac{p(i,j)}{i^2 j^2}$$

74. **Short Run High Gray Level Emphasis (SRHGE)**

$$SRHGE = \frac{1}{N_s}\sum_{i=1}^{N_g}\sum_{j=1}^{N_r} \frac{p(i,j)i^2}{j^2}$$

75. **Long Run Low Gray Level Emphasis (LRLGE)**

$$LRLGE = \frac{1}{N_s}\sum_{i=1}^{N_g}\sum_{j=1}^{N_r} \frac{p(i,j)j^2}{i^2}$$

76. Long Run High Gray Level Emphasis (LRHGE)

$$LRHGE = \frac{1}{N_s} \sum_{i=1}^{N_g} \sum_{j=1}^{N_r} p(i,j) i^2 j^2$$

77. Gray level variance (GLV)

$$GLV = \sum_{i=1}^{N_g} \sum_{j=1}^{N_r} (i - \mu_g)^2 p_n(i,j)$$

78. Run length variance (RLV)

$$RLV = \sum_{i=1}^{N_g} \sum_{j=1}^{N_r} (j - \mu_r)^2 p_n(i,j)$$

79. Run entropy (RE) (8)

$$RE = -\sum_{i=1}^{N_g} \sum_{j=1}^{N_r} p_n(i,j|\theta) \log_2 [p_n(i,j)]$$

Gray-Level size-zone matrix based features

Gray-level size-zone matrix (GLSZM) based features, as described by Thibault et al. (9, 10). A gray level size-zone matrix describes the amount of homogeneous connected areas within the volume, of a certain size and intensity. The (i,j)th entry of the GLSZM $p(i,j)$ is the number of connected areas of gray-level (i.e. intensity value) i and size j. GLSZM features therefore describe homogeneous areas within the tumor volume, describing tumor heterogeneity at a regional scale (11). Let:

$p(i,j)$ be the (i,j)th entry in the given GLSZM p,

N_g the number of discrete intensity values in the image,

N_z the size of the largest, homogeneous region in the volume of interest,

N_s the total number of homogeneous regions (zones), where $N_s = \sum_{i=1}^{N_g} \sum_{j=1}^{N_z} p(i,j)$,

p_z the sum distribution of the number of zones with size j, where $p_z(j) = \sum_{i=1}^{N_g} p(i,j)$,

p_g the sum distribution of the number of zones with gray level i, where $p_g(i) = \sum_{j=1}^{N_z} p(i,j)$,

N_p the number of voxels in the image, where $N_p = \sum_{j=1}^{N_z} j p_z$,

$p_n(i,j)$ the normalized size-zone matrix, where $p_n(i,j) = \frac{p(i,j)}{N_s}$,

μ_z the mean zone size, where $\mu_z = \sum_{i=1}^{N_g} \sum_{j=1}^{N_z} j p_n(i,j|\theta)$,

μ_g the mean gray level, where $\mu_g = \sum_{i=1}^{N_g} \sum_{j=1}^{N_z} i p_n(i,j|\theta)$.

80. Small area Emphasis (SAE)

$$SAE = \frac{1}{N_s} \sum_{j=1}^{N_z} \frac{p_z}{j^2}$$

81. Large area Emphasis (LAE)

$$LAE = \frac{1}{N_s} \sum_{j=1}^{N_z} j^2 p_z$$

82. Intensity Non-Uniformity (IN)

$$IN = \frac{1}{N_s} \sum_{i=1}^{N_g} p_g^2$$

83. **Intensity Non-Uniformity Normalized (INN)**

$$INN = \frac{1}{N_s^2}\sum_{i=1}^{N_g} p_g^2$$

84. **Size-zone Non-Uniformity (SZN)**

$$SZN = \frac{1}{N_s}\sum_{j=1}^{N_z} p_z^2$$

85. **Size-zone Non-Uniformity Normalized (SZNN)**

$$SZNN = \frac{1}{N_s^2}\sum_{j=1}^{N_z} p_z^2$$

86. **Zone Percentage (ZP)**

$$ZP = \frac{N_s}{N_p}$$

87. **Low intensity Emphasis (LIE)**

$$LIE = \frac{1}{N_s}\sum_{i=1}^{N_g} \frac{p_g}{i^2}$$

88. **High intensity Emphasis (HIE)**

$$HIE = \frac{1}{N_s}\sum_{i=1}^{N_g} i^2 p_g$$

89. **Low intensity small area Emphasis (LISAE)**

$$LISAE = \frac{1}{N_s}\sum_{i=1}^{N_g}\sum_{j=1}^{N_z} \frac{p(i,j)}{i^2 j^2}$$

90. **High intensity small area Emphasis (HISAE)**

$$HISAE = \frac{1}{N_s}\sum_{i=1}^{N_g}\sum_{j=1}^{N_z} \frac{p(i,j)i^2}{j^2}$$

91. **Low intensity large area Emphasis (LILAE)**

$$LILAE = \frac{1}{N_s}\sum_{i=1}^{N_g}\sum_{j=1}^{N_z} \frac{p(i,j)j^2}{i^2}$$

92. High intensity large area Emphasis (HILAE)

$$HILAE = \frac{1}{N_s} \sum_{i=1}^{N_g} \sum_{j=1}^{N_z} p(i,j) i^2 j^2$$

93. Intensity variance (IV)

$$IV = \sum_{i=1}^{N_g} \sum_{j=1}^{N_z} (i - \mu_g)^2 p_n(i,j)$$

94. Size-zone variance (SZV)

$$SZV = \sum_{i=1}^{N_g} \sum_{j=1}^{N_z} (j - \mu_z)^2 p_n(i,j)$$

95. Zone entropy (ZE)

$$ZE = -\sum_{i=1}^{N_g} \sum_{j=1}^{N_z} p_n(i,j) \log_2 [p_n(i,j)]$$

Gray-Level distance-zone matrix based features

Gray-level distance-zone matrix (GLDZM) based features, as described by Thibault et al. (12). A gray level distance-zone matrix describes the amount of homogeneous connected areas within the volume, of a certain intensity and distance to the shape border. The shape border is defined by 6-connectedness in 3D (i.e. a voxel is on the border, if at least one face is exposed). In contrast to the original definition by Thibault et al. (12), the minimum distance to the border is 1, instead of 0 (i.e. voxels on the border have a distance of 1), to allow for correct feature calculations. The (i,j)th entry of the GLDZM $p(i,j)$ is the number of connected areas of gray-level (i.e. intensity value) i and minimum distance j to the shape border. GLSZM features therefore describe the radial distribution of homogeneous areas within the tumor volume. Let:

$p(i,j)$ be the (i,j)th entry in the given GLDZM p,

N_g the number of discrete intensity values in the image,

N_d the largest distance of a homogeneous region in the volume of interest to the shape border,

N_s the total number of homogeneous regions (zones), where $N_s = \sum_{i=1}^{N_g} \sum_{j=1}^{N_d} p(i,j)$,

p_d the sum distribution of the number of zones with distance j, where $p_z(j) = \sum_{i=1}^{N_g} p(i,j)$,

p_g the sum distribution of the number of zones with gray level i, where $p_g(i) = \sum_{j=1}^{N_z} p(i,j)$,

N_p the number of voxels in the image, where $N_p = \sum_{j=1}^{N_d} jp_d$,

$p_n(i,j)$ the normalized size-zone matrix, where $p_n(i,j) = \frac{p(i,j)}{N_s}$,

μ_d the mean distance, where $\mu_d = \sum_{i=1}^{N_g} \sum_{j=1}^{N_d} jp_n(i,j|\theta)$,

μ_g the mean gray level, where $\mu_g = \sum_{i=1}^{N_g} \sum_{j=1}^{N_d} ip_n(i,j|\theta)$.

96. Small distance Emphasis (SDE)

$$SDE = \frac{1}{N_s} \sum_{j=1}^{N_d} \frac{p_d}{j^2}$$

97. Large distance Emphasis (LDE)

$$LDE = \frac{1}{N_s}\sum_{j=1}^{N_d} j^2 p_d$$

98. Intensity Non-Uniformity (IN)

$$IN = \frac{1}{N_s}\sum_{i=1}^{N_g} p_g^2$$

99. Intensity Non-Uniformity Normalized (INN)

$$INN = \frac{1}{N_s^2}\sum_{i=1}^{N_g} p_g^2$$

100. Distance-zone Non-Uniformity (DZN)

$$DZN = \frac{1}{N_s}\sum_{j=1}^{N_d} p_d^2$$

101. Distance-zone Non-Uniformity Normalized (DZNN)

$$DZNN = \frac{1}{N_s^2}\sum_{j=1}^{N_d} p_d^2$$

102. Zone Percentage (ZP)

$$ZP = \frac{N_s}{N_p}$$

103. Low intensity Emphasis (LIE)

$$LIE = \frac{1}{N_s}\sum_{i=1}^{N_g} \frac{p_g}{i^2}$$

104. High intensity Emphasis (HIE)

$$HIE = \frac{1}{N_s}\sum_{i=1}^{N_g} i^2 p_g$$

105. Low intensity small distance Emphasis (LISDE)

$$LISAE = \frac{1}{N_s}\sum_{i=1}^{N_g}\sum_{j=1}^{N_d} \frac{p(i,j)}{i^2 j^2}$$

106. **High intensity small distance Emphasis (HISDE)**

$$HISAE = \frac{1}{N_s} \sum_{i=1}^{N_g} \sum_{j=1}^{N_d} \frac{p(i,j) i^2}{j^2}$$

107. **Low intensity large distance Emphasis (LILDE)**

$$LILAE = \frac{1}{N_s} \sum_{i=1}^{N_g} \sum_{j=1}^{N_d} \frac{p(i,j) j^2}{i^2}$$

108. **High intensity large distance Emphasis (HILDE)**

$$HILAE = \frac{1}{N_s} \sum_{i=1}^{N_g} \sum_{j=1}^{N_d} p(i,j) i^2 j^2$$

109. **Intensity variance (IV)**

$$IV = \sum_{i=1}^{N_g} \sum_{j=1}^{N_d} (i - \mu_g)^2 p_n(i,j)$$

110. **Distance-zone variance (DZV)**

$$SZV = \sum_{i=1}^{N_g} \sum_{j=1}^{N_d} (j - \mu_d)^2 p_n(i,j)$$

111. **Distance-zone entropy (DZE)**

$$DZE = -\sum_{i=1}^{N_g} \sum_{j=1}^{N_d} p_n(i,j) \log_2[p_n(i,j)]$$

Intensity histogram features

Intensity histogram features describe the distribution of grey values within the volume, after discretization into intensity level bins was applied. Let:

$X_d = \{X_{d,1}, X_{d,2}, \dots, X_{d,N_v}\}$ be the set of discretized intensity values of the N_v voxels in the volume of interest,

$H = \{n_1, n_2, \dots\}$ be the histogram with frequency count n_i of each discretized intensity level i in X_d,

N_g be the number of discretized intensity values (bins) in the volume of interest,

p_i be the occurrence probability for each bin i of the histogram N_g, where $p_i = n_i/N_v$.

112. Coefficient of variance (cov)

$$cov = \frac{\text{standard deviation}}{\text{mean}}$$

113. Energy

$$energy = \sum_{j=1}^{N_v} X_d(j)^2$$

Energy is also known as the sum of squares.

114. Entropy

$$entropy = -\sum_{i=1}^{N_g} P(i) \log_2 P(i)$$

115. Interquartile range (iqr)

$$IQR = P_{75} - P_{25}$$

where P_{25} and P_{75} are the 25th and 75th percentile of X_d, respectively.

116. Kurtosis

$$kurtosis = \frac{\frac{1}{N_v}\sum_{j=1}^{N_v}(X_d(j) - \bar{X}_d)^4}{\left(\frac{1}{N_v}\sum_{j=1}^{N_v}(X_d(j) - \bar{X}_d)^2\right)^2}$$

where $\bar{X}_d$ is the mean of X_d.

117. Maximum

The maximum discretized intensity value of X_d.

$$maximum = \max(X_d)$$

118. Maximum histogram gradient (maxgrad)

$$maxgrad = \max(H')$$

Where H' is the histogram gradient, defined as:

$$H' = \{H(2) - H(1), \dots, \frac{H(i+1) - H(i-1)}{2}, \dots, H(N_g) - H(N_g - 1)\}$$

119. Maximum histogram gradient intensity level (maxgradi)

The discretized intensity level i corresponding to the maximum histogram gradient.

120. Mean

The mean discretized intensity value of X_d.

$$mean = \frac{1}{N_v}\sum_{j=1}^{N_v} X_d(j)$$

121. Mean absolute deviation (meand)

The mean of the absolute deviations of all discretized intensity levels around the mean of X_d.

$$meand = \frac{1}{N_v}\sum_{j=1}^{N_v}|X_d(j) - \overline{X_d}|$$

where $\bar{X}_d$ is the mean of X_d.

122. Median

The sample median of X_d or the 50th percentile of X_d.

123. Median absolute deviation (mediand)

The dispersion from the median of X_d.

$$mediand = \frac{1}{N_v}\sum_{j=1}^{N_v}|X_d(j) - M|$$

where M is the median of X_d.

124. Minimum

The minimum discretized intensity value of X_d.

$$minimum = \min(X_d)$$

125. Minimum histogram gradient (mingrad)

$$mingrad = \min(H')$$

Where H' is the histogram gradient, defined as:

$$H' = \{H(2) - H(1), \dots, \frac{H(i+1) - H(i-1)}{2}, \dots, H(N_g) - H(N_g - 1)\}$$

126. Minimum histogram gradient intensity level (mingradi)

The discretized intensity level i corresponding to the minimum histogram gradient.

127. **Mode**

The mode of X_d is the most frequently occurring discretized image level present. In case multiple bins have the highest count n_i, the mode is the smallest of those values.

128. **Uniformity**

$$uniformity = \sum_{i=1}^{N_g} P(i)^2$$

129. **Range**

The range of bins in the histogram, i.e. the width of the histogram.

$$range = \max(X_d) - \min(X_d)$$

130. **Root mean square (RMS):**

$$RMS = \sqrt{\frac{\sum_{j=1}^{N_v} X_d(j)^2}{N_v}}$$

131. **Robust mean absolute deviation (rmeand)**

Similar to mean absolute deviation, but in this case only considering the set of intensity levels in the range between the 10th and 90th percentile of X_d.

$$rmeand = \frac{1}{N_{10-90}} \sum_{j=1}^{N_{10-90}} \left| X_{d,10-90}(j) - \bar{X}_{d,10-90} \right|$$

where X_{10-90} represents the set of N_{10-90} voxels in X_d whose discretized intensity levels fall within the range of the 10th till the 90th percentile of X_d.

132. **Skewness**

$$skewness = \frac{\frac{1}{N_v}\sum_{j=1}^{N_v}(X_d(j) - \bar{X}_d)^3}{\left(\sqrt{\frac{1}{N_v}\sum_{j=1}^{N_v}(X_d(j) - \bar{X}_d)^2}\right)^3}$$

133. Standard deviation

$$standard\ deviation = \left(\frac{1}{N_v - 1}\sum_{j=1}^{N_v}(X_d(j) - \bar{X}_d)^2\right)^{1/2}$$

134. Variance

The variance of X_d.

$$variance = \frac{1}{N_v - 1}\sum_{j=1}^{N_v}(X_d(j) - \overline{X_d})^2$$

where $\overline{X_d}$ is the mean of X_d.

135. 10th percentile

The 10th percentile of X_d.

136. 90th percentile

The 90th percentile of X_d.

137. Quartile coefficient of dispersion (qcod)

The quartile coefficient of dispersion is a robust alternative to the coefficient of variance.

$$qcod = \frac{P_{75} - P_{25}}{P_{75} + P_{25}}$$

where P_{25} and P_{75} are the 25th and 75th percentile of X_d, respectively.

Intensity volume histogram (IVH) features (PET images only)

A set of metrics derived from intensity volume histogram (IVH) representations (13), which summarize the complex three dimensional (3D) data contained in the image into a single curve, allowing for a simplified interpretation. The following definitions of IVH features were used:

138. ***$AVAI_y$***
Volume (AV) [ml] above (i.e. with at least) an intensity (AI)

139. ***$RVAI_y$***
Relative volume (RV) [%] above (i.e. with at least) an intensity (AI)

140. ***$AVRI_x$***
Volume (AV) [ml] above (i.e. with at least) a relative intensity (RI)

141. ***$RVRI_x$***
Relative volume (RV) [%] above (i.e. with at least) a relative intensity (RI)

142. ***$AIAV_z$***
Intensity thresholds (AI) [SUV] for the Z ml highest intensity volume (AV)

143. ***$AIRV_x$***
Intensity thresholds (AI) [SUV] for the X% highest intensity volume (RV)

144. ***$MIAV_z$***
Mean intensity (MI) [SUV] in the Z ml highest intensity volume (AV)

145. ***$MIRV_x$***
Mean intensity (MI) [SUV] in the X% highest intensity volume (RV)

146. ***$TLGAI_y$***
TLG for volume (TLG) above (i.e. with at least) an intensity (AI)

147. ***$TLGRI_x$***
TLG for volume (TLG) above (i.e. with at least) a relative intensity (RI)

Relative steps in volume and intensity (**x**) are taken in 10% increments; X={10%, 20%,…, 90%}. Absolute steps in intensity (**y**) are taken in absolute [SUV] increments, e.g. 0.5; Y={0.5, 1,…, SUV_{max}}, where SUV_{max} is the maximum image intensity value. Absolute steps in volume (**z**) are taken in 0.5 ml increments; Z={0.5 ml, 1 ml,…, V}, where V is the tumor volume.

Local Intensity features

Local Intensity (LocInt) features are defined based on local intensity values around a center voxel.

148. Local intensity peak

Mean intensity level in a 1 cm^3 spherical volume, centered on the voxel with the maximum intensity level in the volume of interest. In case multiple voxels contain the maximum intensity level, the highest mean intensity level of all spherical volumes is used.

149. Global Intensity peak

Similar to local intensity peak, but in this case the mean intensity level in a 1 cm^3 spherical volume is calculated for every voxel in the volume of interest. The highest mean intensity level of all spherical volumes is selected as the global intensity peak feature.

Neighborhood gray tone difference matrix based features

Neighborhood gray tone difference matrix (NGTDM) based features, as described by Amadasun and King (14). The ith entry of the NGTDM $s(i|d)$ is the sum of gray level differences of voxels with intensity i and the average intensity A_i of their neighboring voxels within a distance d. In contrast to the original paper, a complete neighborhood is not required and A_i is determined over the valid voxels. Let:

n_i be the number of voxels with gray level i,

$N_v = \sum n_i$, the total number of voxels (defined as n^2 by Amadasun and King (14)),

$s(i) = \begin{cases} \sum_{n_i} |i - A_i| & \text{for } n_i > 0 \\ 0 & \text{otherwise} \end{cases}$, generalized for any distance d,

N_g be the maximum discrete intensity level in the image,

$p(i) = \frac{n_i}{N_v}$, the probability of gray level i,

N_p, the total number of gray levels present in the image.

150. Coarseness

$$coarseness = \frac{1}{\varepsilon + \sum_{i=i}^{N_g} p(i)s(i)}$$

Where ε is a small number to prevent coarseness becoming infinite.

151. Contrast

$$contrast = \left(\frac{1}{N_p(1 - N_p)} \sum_{i=1}^{N_g} \sum_{j=1}^{N_g} p(i)p(j)(i-j)^2 \right) \left(\frac{1}{N_v} \sum_{i=i}^{N_g} s(i) \right)$$

152. Busyness

$$busyness = \frac{\sum_{i=i}^{N_g} p(i)s(i)}{\sum_{i=i}^{N_g} \sum_{j=i}^{N_g} |ip(i) - jp(j)|}, \qquad p(i) \neq 0,\ p(j) \neq 0$$

153. Complexity

$$complexity = \frac{1}{N_v} \sum_{i=i}^{N_g} \sum_{j=i}^{N_g} |i-j| \frac{p(i)s(i) + p(j)s(j)}{p(i) + p(j)}, \qquad p(i) \neq 0,\ p(j) \neq 0$$

154. Strength

$$strength = \frac{\sum_{i=i}^{N_g} \sum_{j=i}^{N_g} [p(i) + p(j)](i-j)^2}{\varepsilon + \sum_{i=i}^{N_g} s(i)}, \qquad p(i) \neq 0,\ p(j) \neq 0$$

Neighboring gray level dependence matrix based features

Neighboring gray level dependence matrix (NGLDM) based features, as described by Sun and Wee (15). NGLDM features are invariant under spatial rotation. The (i,j)th entry of the NGLDM $p(i,j|d,a)$ describes the number of neighborhoods with center voxel gray-level (i.e. intensity value) i and dependence (i.e. number of dependent voxels) $k = j - 1$. A neighborhood are all voxels within a distance d from the center voxel. The center voxel and a neighboring voxel are dependent if their absolute gray value difference $\leq a$, the dependency coarseness parameter. The features originally specified by Sun and Wee are analogous to the GLRLM and GLSZM features, and the feature set is extended accordingly. Let:

$p(i,j)$ be the (i,j)th entry in the given NGLDM p, generalized for any d and a,

N_g the number of discrete intensity values in the image,

N_d the maximum dependence value,

N_s the total number of neighborhoods, where $N_s = \sum_{i=1}^{N_g} \sum_{j=1}^{N_z} p(i,j)$,

p_d the sum distribution of the number of neighborhoods with dependence $j = k + 1$, where $p_d(j) = \sum_{i=1}^{N_g} p(i,j)$,

p_g the sum distribution of the number of neighborhoods with center voxel gray level i, where $p_g(i) = \sum_{j=1}^{N_d} p(i,j)$,

$p_n(i,j)$ the normalized NGLDM, where $p_n(i,j) = \frac{p(i,j)}{N_s}$,

μ_d the mean dependence, where $\mu_r = \sum_{i=1}^{N_g} \sum_{j=1}^{N_z} j p_n(i,j|\theta)$,

μ_g the mean gray level, where $\mu_g = \sum_{i=1}^{N_g} \sum_{j=1}^{N_z} i p_n(i,j|\theta)$.

Note: By definition, the number of voxels in the image (N_p) equals the total number of neighborhoods (N_s), since in our implementation every voxel is considered to have a neighborhood. Feature “dependence percentage” $\left(\frac{N_s}{N_p}\right)$, which is the equivalent to run-length feature “run percentage” (RP; 70), is therefore omitted, because it will always evaluate to 1.

155. Small Dependence Emphasis (SDE)

$$SDE = \frac{1}{N_s} \sum_{j=1}^{N_d} \frac{p_d}{j^2}$$

This feature is also called Small Number Emphasis (15).

156. Large Dependence Emphasis (LDE)

$$LDE = \frac{1}{N_s} \sum_{j=1}^{N_d} j^2 p_d$$

This feature is also called Large Number Emphasis (15).

157. Gray-level Non-Uniformity (GLN)

$$GLN = \frac{1}{N_s} \sum_{i=1}^{N_g} p_g^2$$

158. Gray-level Non-Uniformity Normalized (GLNN)

$$GLNN = \frac{1}{N_s^2} \sum_{i=1}^{N_g} p_g^2$$

159. Dependence Non-Uniformity (DN)

$$DN = \frac{1}{N_s} \sum_{j=1}^{N_d} p_z^2$$

This feature is also called Number Nonuniformity (15).

160. Dependence Non-Uniformity Normalized (DNN)

$$DNN = \frac{1}{N_s^2} \sum_{j=1}^{N_z} p_z^2$$

161. Low Gray-level Emphasis (LGE)

$$LGE = \frac{1}{N_s} \sum_{i=1}^{N_g} \frac{p_g}{i^2}$$

162. High Gray-level Emphasis (HGE)

$$HGE = \frac{1}{N_s} \sum_{i=1}^{N_g} i^2 p_g$$

163. Low Gray-level small Dependence Emphasis (LGSDE)

$$LGSDE = \frac{1}{N_s} \sum_{i=1}^{N_g} \sum_{j=1}^{N_d} \frac{p(i,j)}{i^2 j^2}$$

164. High Gray-level small Dependence Emphasis (HGSDE)

$$HGSDE = \frac{1}{N_s} \sum_{i=1}^{N_g} \sum_{j=1}^{N_d} \frac{p(i,j) i^2}{j^2}$$

165. Low Gray-level large Dependence Emphasis (LGLDE)

$$LGLDE = \frac{1}{N_s} \sum_{i=1}^{N_g} \sum_{j=1}^{N_d} \frac{p(i,j) j^2}{i^2}$$

166. High Gray-level large Dependence Emphasis (HGLDE)

$$HGLDE = \frac{1}{N_s} \sum_{i=1}^{N_g} \sum_{j=1}^{N_d} p(i,j) i^2 j^2$$

167. Gray-level variance (GLV)

$$GLV = \sum_{i=1}^{N_g} \sum_{j=1}^{N_d} (i - \mu_g)^2 p_n(i,j)$$

168. Dependence variance (DV)

$$DV = \sum_{i=1}^{N_g} \sum_{j=1}^{N_d} (j - \mu_d)^2 p_n(i,j)$$

169. Dependence entropy (DE), also called Entropy (15)

$$DE = -\sum_{i=1}^{N_g} \sum_{j=1}^{N_d} p_n(i,j) \log_2 [p_n(i,j)]$$

Note: the definition of entropy by Sun and Wee (15) uses the dependence counts (p) instead of the dependence propabilities (p_n).

170. Second moment (SM) (15)

$$SM = \frac{\sum_{i=1}^{N_g} \sum_{j=1}^{N_d} p(i,j)^2}{N_s}$$

Note: for this feature, defined by Sun and Wee (15), there is no gray-level run-length equivalent.

Radial gradient and radial deviation features (CT images only)

NB: the current implementation is a beta version.

Radial gradient and radial deviation features, as described by Tunali et al. (16). First, a radial gradient map and a radial deviation map is derived from image. For each voxel in the volume of interest, a radial deviation and a radial gradient value is determined, which results in radial gradient and radial deviation maps. Each voxel in the radial deviation map is defined as the angle between the gradient vector of a voxel and its radial vector, which points towards the center of mass of the VOI. Each voxel in the radial gradient map represents the magnitude of the gradient along the radial vector of that voxel. From the radial gradient and radial deviation maps, the following features are calculated:

171. **Radial deviation tumor mean**
172. **Radial deviation tumor SD**
173. **Radial gradient tumor mean**
174. **Radial gradient tumor SD**
175. **Radial deviation tumor mean (2D)**
176. **Radial deviation tumor SD (2D)**
177. **Radial gradient tumor mean (2D)**
178. **Radial gradient tumor SD (2D)**
179. **Radial deviation core mean**
180. **Radial deviation core SD**
181. **Radial gradient core mean**
182. **Radial gradient core SD**
183. **Radial deviation core mean (2D)**
184. **Radial deviation core SD (2D)**
185. **Radial gradient core mean (2D)**
186. **Radial gradient core SD (2D)**
187. **Radial deviation border mean**
188. **Radial deviation border SD**
189. **Radial gradient border mean**
190. **Radial gradient border SD**
191. **Radial deviation border mean (2D)**
192. **Radial deviation border SD (2D)**
193. **Radial gradient border mean (2D)**
194. **Radial gradient border SD (2D)**
195. **Radial deviation outside mean**
196. **Radial deviation outside SD**
197. **Radial gradient outside mean**
198. **Radial gradient outside SD**
199. **Radial deviation outside mean (2D)**
200. **Radial deviation outside SD (2D)**
201. **Radial gradient outside mean (2D)**
202. **Radial gradient outside SD (2D)**
203. **Radial deviation outside-tumor separation mean**
204. **Radial deviation outside-tumor separation SD**
205. **Radial gradient outside-tumor separation mean**
206. **Radial gradient outside-tumor separation SD**
207. **Radial deviation outside-tumor separation mean (2D)**

208. Radial deviation outside-tumor separation SD (2D)
209. Radial gradient outside-tumor separation mean (2D)
210. Radial gradient outside-tumor separation SD (2D)
211. Radial deviation outside-border separation mean
212. Radial deviation outside-border separation SD
213. Radial gradient outside-border separation mean
214. Radial gradient outside-border separation SD
215. Radial deviation outside-border separation mean (2D)
216. Radial deviation outside-border separation SD (2D)
217. Radial gradient outside-border separation mean (2D)
218. Radial gradient outside-border separation SD (2D)

Filtered features

Wavelet features

Wavelet transform effectively decouples textural information by decomposing the original image, in a similar manner as Fourier analysis, in low– and high-frequencies. A (undecimated) three dimensional wavelet transform decomposes the original image X into 8 decompositions (**Figure 1**). Consider L and H to be a low-pass (i.e. a scaling) and, respectively, a high-pass (i.e. a wavelet) function, and the wavelet decompositions of X to be labeled as X_{LLL}, X_{LLH}, X_{LHL}, X_{LHH}, X_{HLL}, X_{HLH}, X_{HHL} and X_{HHH}. For example, X_{LLH} is then interpreted as the high-pass sub band, resulting from directional filtering of X with a low-pass filter along x-direction, a low pass filter along y-direction and a high-pass filter along z-direction and is constructed as:

$$X_{LLH}(i,j,k) = \sum_{p=1}^{N_L}\sum_{q=1}^{N_L}\sum_{r=1}^{N_H} L(p)L(q)H(r)X(i+p,j+q,k+r)$$

Where N_L is the length of filter L and N_H is the length of filter H. The other decompositions are constructed in a similar manner, applying their respective ordering of low or high-pass filtering in x, y and z-direction. If the applied wavelet decomposition is undecimated, the size of each decomposition is equal to the original image and each decomposition is shift invariant. Because of these properties, original (tumor) segmentations can be applied directly to the decompositions after wavelet transform. In previous studies, the “Coiflet 1” wavelet was applied (2, 17-20). For each decomposition, for instance, first order gray level statistics and the textural features can be computed.

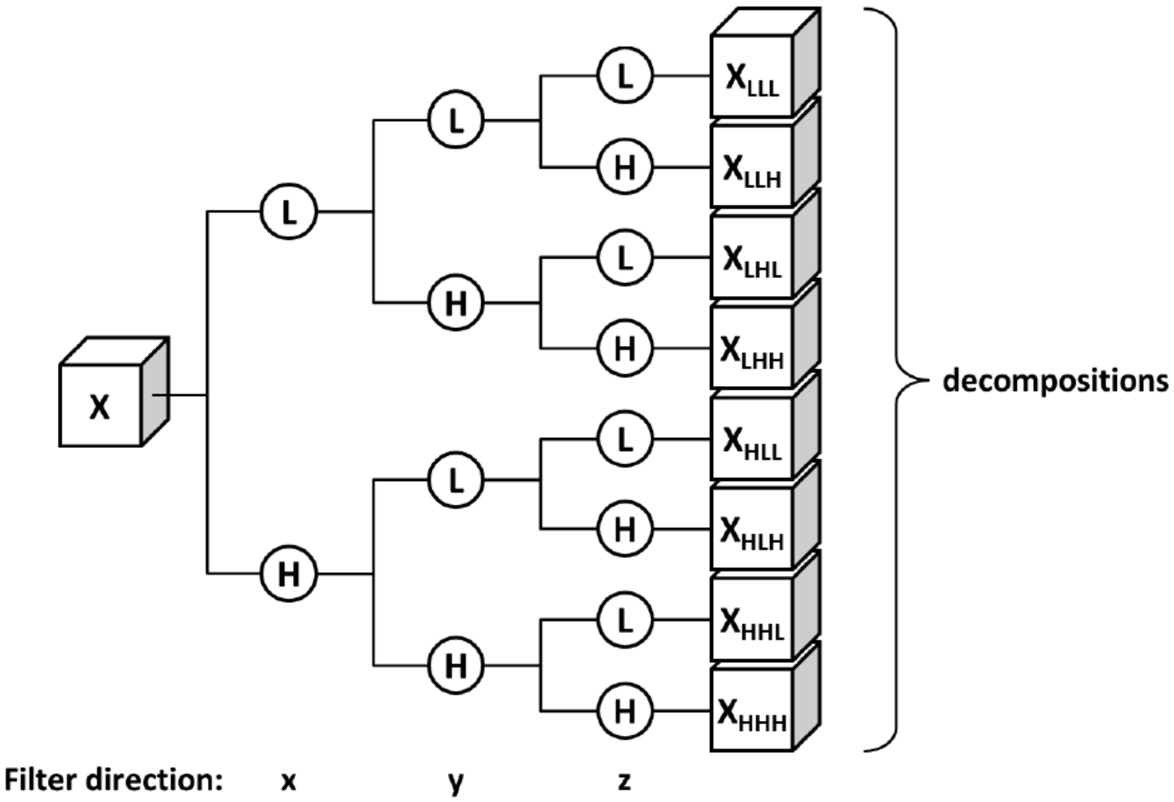

Figure 1: *Schematic of the undecimated three dimensional wavelet transform. The original image* X *is decomposed into 8 decompositions, by directional low-pass (i.e. a scaling) and high-pass (i.e. a wavelet) filtering:* X_{LLL}, X_{LLH}, X_{LHL}, X_{LHH}, X_{HLL}, X_{HLH}, X_{HHL} *and* X_{HHH}.

Laplacian of Gaussian features

The Laplacian of an image brings out areas of rapid intensity change and is usually used for edge detection. A Gaussian filter is applied prior to the Laplacian to smooth the image and reduce noise. Textural properties representing features of different degrees of coarseness can then be calculated. The equation of a Laplacian of Gaussian (LoG) with a 2D kernel:

$$\mathrm{LoG}(x, y) = -\frac{1}{\pi\sigma^4}\left[1 - \frac{x^2 + y^2}{2\sigma^2}\right] e^{-\frac{x^2+y^2}{2\sigma^2}}$$

Texture size (fine to coarse) is highlighted by modifying the Gaussian radius parameter σ (e.g., from 0.5 mm to 5mm, with 0.5 mm increments). Each value of σ provides a filtered image. For instance first-order gray-level statistics (described earlier) can be determined for each filtered image, as well as for only the positive part of each filtered image.

References

1. Al-Kadi OS, ast, Watson D. Texture Analysis of Aggressive and Nonaggressive Lung Tumor CE CT Images. IEEE Transactions on Biomedical Engineering. 2008;55(7):1822-30.
2. Aerts HJ, Velazquez ER, Leijenaar RT, Parmar C, Grossmann P, Carvalho S, et al. Decoding tumour phenotype by noninvasive imaging using a quantitative radiomics approach. Nature Communications. 2014;5:4006.
3. Sousa JR, Silva AC, de Paiva AC, Nunes RA. Methodology for automatic detection of lung nodules in computerized tomography images. Comput Methods Programs Biomed. 2010;98(1):1-14.
4. Haralick RM, Shanmugam K, Dinstein I. Textural Features of Image Classification. IEEE T Syst Man Cyb. 1973;SMC-3(6):610-21.
5. Clausi DA. An analysis of co-occurrence texture statistics as a function of grey level quantization. Canadian Journal of remote sensing. 2002;28(1):45-62.
6. Tsatsoulis LSaC. Texture Analysis of SAR Sea Ice Imagery Using Gray Level Co-Occurrence Matrices. IEEE Transactions on Geoscience and Remote Sensing. 1999;37(2).
7. Galloway M. Texture analysis using gray level run lengths. Comput Vision Graph. 1975;4:172-9.
8. Albregtsen F, Nielsen B, Danielsen HE, editors. Adaptive gray level run length features from class distance matrices. Pattern Recognition, 2000 Proceedings 15th International Conference on; 2000 2000.
9. Thibault GF, B; Navarro, C; Pereira, S. Texture indexes and gray level size zone matrix: application to cell nuclei classification. Pattern Recognition Inf Process. 2009:140-5.
10. Tixier F, Hatt M, Le Rest CC, Le Pogam A, Corcos L, Visvikis D. Reproducibility of tumor uptake heterogeneity characterization through textural feature analysis in 18F-FDG PET. J Nucl Med. 2012;53(5):693-700.
11. Tixier F, Le Rest CC, Hatt M, Albarghach N, Pradier O, Metges JP, et al. Intratumor heterogeneity characterized by textural features on baseline 18F-FDG PET images predicts response to concomitant radiochemotherapy in esophageal cancer. J Nucl Med. 2011;52(3):369-78.
12. Thibault G, Angulo J, Meyer F. Advanced Statistical Matrices for Texture Characterization: Application to Cell Classification. IEEE Transactions on Biomedical Engineering. 2014;61(3):630-7.
13. El Naqa I, Grigsby P, Apte A, Kidd E, Donnelly E, Khullar D, et al. Exploring feature-based approaches in PET images for predicting cancer treatment outcomes. Pattern Recognit. 2009;42(6):1162-71.
14. Amadasun M, King R. Textural features corresponding to textural properties. Systems, Man and Cybernetics, IEEE Transactions on. 1989;19(5):1264-74.
15. Sun C, Wee WG. Neighboring gray level dependence matrix for texture classification. Computer Vision, Graphics, and Image Processing. 1983;23(3):341-52.
16. Tunali I, Stringfield O, Guvenis A, Wang H, Liu Y, Balagurunathan Y, et al. Radial gradient and radial deviation radiomic features from pre-surgical CT scans are associated with survival among lung adenocarcinoma patients. Oncotarget. 2017;8(56):96013-26.
17. Leijenaar RT, Carvalho S, Hoebers FJ, Aerts HJ, van Elmpt WJ, Huang SH, et al. External validation of a prognostic CT-based radiomic signature in oropharyngeal squamous cell carcinoma. Acta Oncol. 2015;54(9):1423-9.
18. van Timmeren JE, Leijenaar RTH, van Elmpt W, Reymen B, Oberije C, Monshouwer R, et al. Survival prediction of non-small cell lung cancer patients using radiomics analyses of cone-beam CT images. Radiother Oncol. 2017.
19. Larue RTHM, Van De Voorde L, van Timmeren JE, Leijenaar RTH, Berbee M, Sosef MN, et al. 4DCT imaging to assess radiomics feature stability: An investigation for thoracic cancers. Radiother Oncol. 2017.

20. Parmar C, Leijenaar RT, Grossmann P, Rios Velazquez E, Bussink J, Rietveld D, et al. Radiomic feature clusters and prognostic signatures specific for Lung and Head & Neck cancer. Sci Rep. 2015;5:11044.
