# Supplementary material for: [18F]FDG PET radiomics to predict disease-free survival in cervical cancer: a multi-scanner/center study with external validation
Source: Eur J Nucl Med Mol Imaging. 2021 Mar 26;48(11):3432–43. doi: 10.1007/s00259-021-05303-5 (PMC8440288; doi:10.1007/s00259-021-05303-5)
Supplement: Supplementary file 3 — C) Bootstrap mean AUC, F1-score and F2-score, precision, recall and AUCpr values of the best models using ComBat harmonization (PDF 125 kb) [file 259_2021_5303_MOESM3_ESM.pdf]

Supplementary data C

Table 1

Bootstrap mean AUC, F_1-score and F_2-score, precision, recall and AUCpr values of the best models using ComBat harmonization. Table 1.a) shows the best OR and TLR models using ComBat before FS and table 1.b) shows the effect of ComBat in the best obtained OR and TLR models (after FS). The 95% confidence intervals are in parentheses.

a)

	AUC	*F_1-score*	*F_2-score*	*Precision*	*Recall*	*AUCpr*
OR Model	0.64 (0.51-0.71)	0.39 (0.29-0.50)	0.48 (0.34-0.59)	0.29 (0.21-0.43)	0.59 (0.40-0.80)	0.45 (0.26-0.58)
TLR Model	0.63 (0.54-0.72)	0.40 (0.20-0.55)	0.47 (0.30-0.58)	0.32 (0.18-0.50)	0.54 (0.2-0.60)	0.46 (0.27-0.68)

b)

	AUC	*F_1-score*	*F_2-score*	*Precision*	*Recall*	*AUCpr*
OR Model	0.62 (0.43-0.70)	0.40 (0.17-0.78)	0.51 (0.19-0.67)	0.30 (0.14-0.44)	0.62 (0.40-0.80)	0.32 (0.19-0.49)
TLR Model	0.63 (0.49-0.73)	0.38 (0.17-0.57)	0.46 (0.19-0.69)	0.30 (0.14-0.44)	0.53 (0.2-0.80)	0.41 (0.24-0.58)
